# Structural Variants in the Human Genome Associated With Gut Microbiome and Blood Metabolic Traits With Potential Causal Links

**DOI:** 10.1002/advs.78201

**Published:** 2026-10-11

**Authors:** Qun Zhou, Keqing Wang, Junhong Chen, Haoyu Wang, Xinjiang Tan, Xuanlin Huang, Zhiyin Lin, Xun Xu, Xin Jin, Shaoqiao Zhang, Liang Xiao, Yuanqiang Zou, Tao Zhang, Xiaomin Liu

**Affiliations:** ^1^ BGI Research Wuhan China; ^2^ State Key Laboratory of Genome and Multi‐Omics Technologies BGI Research Shenzhen China; ^3^ College of Life Sciences University of Chinese Academy of Sciences Beijing China; ^4^ Shenzhen Key Laboratory of Human Commensal Microorganisms and Health Research BGI Research Shenzhen China; ^5^ School of Public Health and Emergency Management School of Medicine Southern University of Science and Technology Shenzhen China; ^6^ SUSTech Homeostatic Medicine Institute School of Medicine Southern University of Science and Technology Shenzhen China

**Keywords:** blood metabolites, genome‐wide association analysis, gut microbiome, host‐microbe interactions, structural variants

## Abstract

Research on genetic variants affecting the gut microbiome and blood metabolites has mainly focused on single nucleotide variants (SNPs), leaving structural variants (SVs) largely unexplored. Here, we analyzed high‐coverage (∼42×) whole‐genome sequencing data from 2002 individuals and constructed a catalog of 138 859 high‐confidence SVs, including 44.3% variants not represented in public SV resources. Genome‐wide association analyses of 22 519 common SVs identified 30 microbiome‐associated and 48 metabolite‐associated SV loci, including 19 study‐wide significant associations. Notable signals included an Asian‐specific 6.5k deletion at *TENT2* associated with *Coprobacillus sp. 29_1* abundance (β = 0.158, *p* = 2.5 × 10^−9^) and a deletion covering *HBA1/HBA2/HBM/HBQ1* linked to nine blood metabolic traits (strongest for MCV; β = ‐0.570, *p* = 1.22 × 10^−91^). Conditional and variance‐partitioning analyses indicated that SVs capture complementary genetic information beyond tested SNP signals. SV‐based Mendelian randomization delineated putative directional relationships, including *Clostridium ramosum* with serum uric acid and hyperuricemia. In vitro experiments further supported three predicted metabolite–microbe interactions, showing that vitamin K1 inhibited *Coprococcus comes*, hydroxyproline inhibited *Veillonella parvula*, and cysteine promoted *Fusobacterium mortiferum* growth. Together, our study provides a comprehensive SVs resource and highlights SVs as overlooked genetic determinants of host‐microbiome‐metabolome interactions relevant to human physiology and disease.

## Graphical abstract

Based on a multi‐omics cohort of 2002 individuals comprising high‐depth whole‐genome and metagenomic sequencing data, blood metabolites, and detailed questionnaires, we identified 138 859 high‐confidence SVs, uncovering 36 significant SV–microbiome associations and 48 SV–blood metabolite associations. SV‐based Mendelian randomization identified potential causal relationships, and in vitro validation confirms three metabolite–microbe interactions, such as VK1‐mediated inhibition of *Coprococcus comes*. This study provides a detailed SV catalog for the Chinese population and highlights the significant role of SVs in the gut microbiome, blood metabolites, and their interactions.

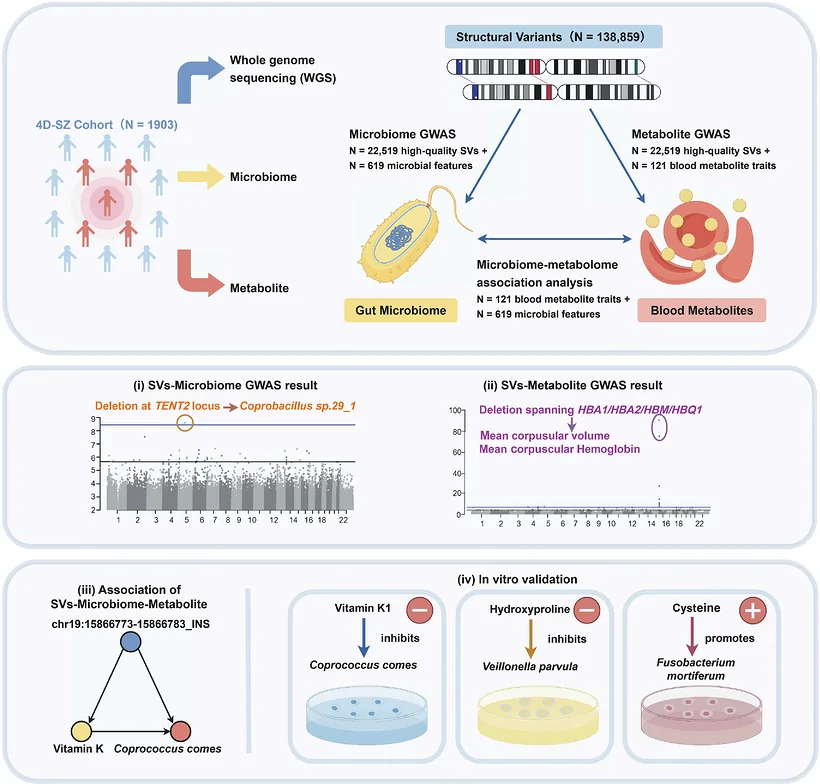

## 1 Introduction

The human gut microbiome plays a pivotal role in numerous physiological processes and is closely linked to human health and the development of diseases such as obesity [1, 2], diabetes [3, 4], inflammatory bowel disease [5, 6], rheumatoid arthritis [7, 8], cardiovascular diseases [9], and mental disorders [10, 11]. Apart from being influenced by factors such as environment, diet, lifestyle, and diseases, the gut microbiome is also controlled to some extent by host genetic factors [12, 13]. In recent years, many microbial genome‐wide association studies (mbGWAS) have identified various host genetic variants that influence the gut microbiome [14, 15, 16, 17, 18], with only the *LCT* and *ABO* loci being frequently discussed and well replicated.

Despite the progress made in understanding the relationship between host genetics and the gut microbiome, most mbGWAS studies have primarily focused on single nucleotide polymorphisms (SNPs) using genotyping arrays, except for the human microbiome project (HMP) and our prior research [19, 20]. This approach has overlooked the potential impact of more extensive and complex genomic variations, such as structural variations (SVs). SVs, which constitute a significant form of genomic variation, include alterations exceeding 50 base pairs (bp) in length, such as deletions (DEL), insertions (INS), inversions (INV), duplications (DUP), and copy number variants (CNV). These SVs are prevalent in the human genome and have been associated with various disease phenotypes [21, 22, 23, 24]. A recent study identified a correlation between the CNV of the human salivary amylase gene (*AMY1*) and the relative abundance of the genus *Ruminococcus* in the gut microbiome [25]. However, this study solely relied on PCR technology to explore the impact of a single human *AMY1* gene copy number variation on the microbiome. Given that few studies have had both whole‐genome and whole‐metagenome data in the same cohort, thus to date, the impact of host SVs on the gut microbiome remains largely unexplored.

Beyond microbial composition itself, blood metabolites represent a critical functional interface through which the gut microbiome exerts systemic effects on host physiology. A growing body of evidence underscores a profound crosstalk between the gut microbiome and the host metabolites [26, 27, 28]. Gut microbes actively participate in the biosynthesis and transformation of a vast array of metabolites, including short‐chain fatty acids, bile acids, vitamins, and tryptophan derivatives, which can enter systemic circulation [29, 30]. These microbially influenced metabolites, in turn, function as key signaling molecules and metabolic regulators, influencing immune responses, energy metabolism, and even brain function via various host receptor‐mediated pathways [31, 32]. Consequently, the interplay between the gut microbiome and blood metabolites is increasingly recognized as a fundamental mechanism underlying host‐microbiome interactions and their impact on health and disease [33, 34, 35]. Measuring the blood metabolites thus provides a powerful, functional readout of host‐microbiome crosstalk.

A critical and underexplored question is how host genetics—particularly structural variations—shapes this functional crosstalk. However, the genome‐wide contribution of host SVs to blood metabolic traits has not been explored. Very limited evidence has been reported. For example, Chen et al. identified a deletion of the *ALB* promoter and a CNV at *PDPR* that were significantly associated with blood metabolic traits in the Finnish population [36]. The NHLBI TOPMed program reported 21 independent SVs or 41 SV‐trait associations across 24 quantitative hematologic traits [37]. While these studies have identified associations between SVs and metabolic traits in Finnish and European populations, there is a notable gap in research focusing on Asian populations. Recent efforts to address this gap include a study using long‐read sequencing in a Chinese population, which identified 22 SVs on 14 chromosomes associated with 13 phenotypes [38]. However, the association analysis was conducted on a maximum of 327 individuals. This underscores the need for a comprehensive investigation of SV contributions to blood metabolic traits in larger Asian populations. Moreover, while existing studies have largely examined host genetics, the microbiome, and metabolites in isolation, a critical gap remains in understanding how host SVs may concurrently contribute to variation in both the gut microbiome and the systemic metabolome within the same population. Comprehensively excavating this tripartite relationship using integrated multi‐omics data from a well‐designed cohort is therefore essential to advance a mechanistic understanding of host‐microbiome interactions.

To address the research gap and investigate the role of SVs in the gut microbiome and blood metabolic traits, we constructed and characterized a high‐quality SV profile of the Chinese population based on 2002 individuals from the 4D‐SZ cohort [19, 20, 39, 40, 41], a multi‐omics cohort comprising high‐depth whole‐genome and metagenomics sequencing, blood metabolites, and detailed questionnaires. Subsequently, we conducted genome‐wide association analyses to identify common SVs and genes harboring rare SVs that significantly contributed to gut microbial taxa/pathways and blood metabolic traits, respectively. Moreover, we examined intricate relationships between the gut microbiome and blood metabolites through their associated SVs as mediators (see Graphic abstract). The primary objective of this study was to characterize the landscape of SVs in the Chinese population and identify significant SVs associated with the microbiome and metabolites, while offering valuable insights into the mechanisms governing host‐microbiome interactions.

## 2 Results

### 2.1 Characterization of SVs Identified in the Chinese Population

We utilized high‐depth whole‐genome sequencing (WGS) data to identify SVs in 2002 Chinese individuals from the 4D‐SZ cohort. Among the sequenced samples, 53% were females, with an average age of 30 years ranging from 21 to 67 years (Figure S1). The mean sequencing depth per individual was 42×, amounting to an average of 122.46 Gb of sequenced data (Figure S2a). The base mapping rate varied from 96.74% to 100%, with an average of up to 99.89% (Figure S2b). An advanced Genome Analysis Toolkit's Structural Variant (GATK‐SV) pipeline [42, 43] was employed to detect SVs (Figure S3; Methods). Following rigorous quality control measures for both samples and variants, a total of 1839 samples were retained. We detected a total of 138 859 high‐confidence SVs with quality scores surpassing 250 or marked as “PASS,” while excluding “BND” variant types (Table S1). These SVs comprised 73 288 insertions (INSs), 38 782 deletions (DELs), 25 030 duplications (DUPs), 1153 complex SVs (CPXs), 378 inversions (INVs), and 228 copy number variations (CNVs) (Figure 1a). The most prevalent SV type was INSs, accounting for 52.78% of all SVs, followed by DELs, representing 27.93% (Figure 1a).

**FIGURE 1:**
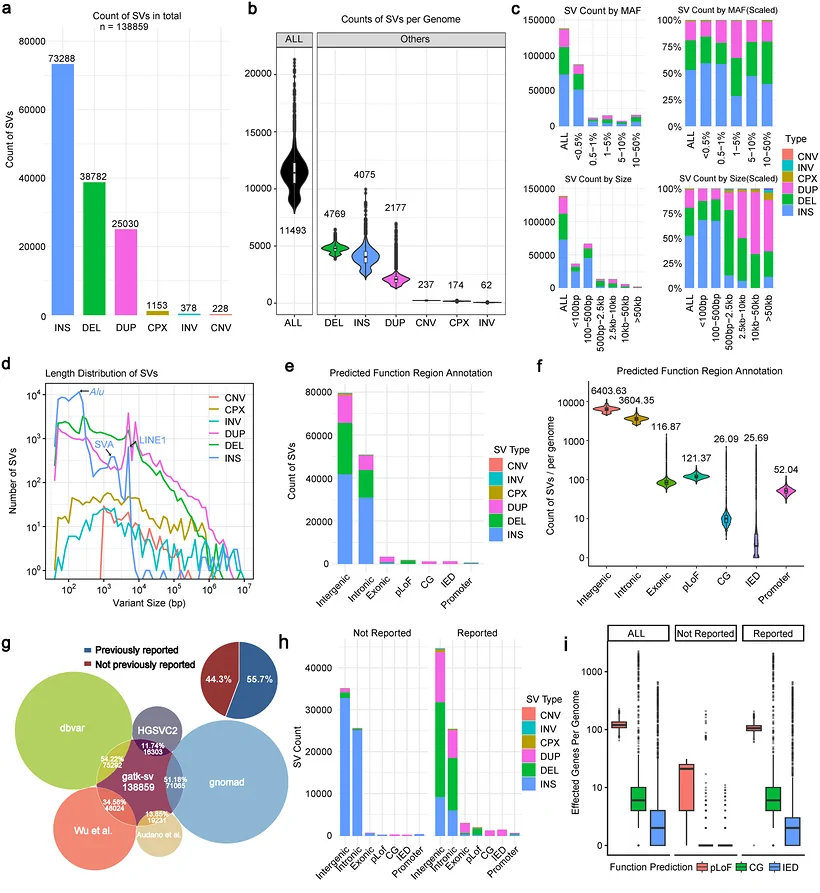
Characteristics of the SVs discovered in this study (4D‐SZ Cohort). (a) Descriptive statistics of the SVs count for each SV type. (b) The count of SVs per sample by each SV type. (c) SV count distribution by minor allele frequency (MAF) and size. (d) Length distribution of SVs. The peaks represent the Alu, SVA, and LINE1 events. (e) The count of SVs according to the predicted function region annotation by each SV type. (f) The count of SVs per sample according to the predicted function region annotation by each SV type. (g) Venn diagram showing the overlap between SVs in this study and published SV datasets. Among the SVs, 55.7% have been previously reported, while 44.3% were not previously reported. (h) The difference in SV counts between reported and not reported SVs according to the predicted function region annotation. (i) The counts of affected genes per genome by reported and not reported SVs according to the predicted function region annotation. Differences between reported and not reported SVs were assessed with two‐sided *t*‐tests. CPXs were not included in this analysis due to a lack of accurate annotation of the genes they affected. Abbreviations: SV, structural variant; DEL, deletions; DUP, duplications; INS, insertions; INV, inversions; CPX, complex variants; CNV, copy number variation.

Despite the overall higher count of INSs than DELs in the population, each individual exhibited a greater number of DELs than INSs, consistent with an earlier study [44]. On average, each genome harbored 11 493 SVs events (ranging from 7851 to 21 324), exceeding the SV count documented in the 1000 genome phase3 (9679 SVs; average depth of 30×) and the Genome Aggregation Database (gnomAD [42]; 7439 SVs; average depth of 32×), yet lower than a long‐read sequencing study [38] (18 489 SVs; average depth of 17×). DELs and INSs remained the most prevalent SV types, constituting 41.5% and 35.5% of all identified SVs, respectively. The average numbers of DELs, INSs, DUPs, CNVs, CPXs, and INVs per sample were 4769, 4075, 2177, 237, 174, and 62, respectively (Figure 1b).

To evaluate the identified SVs, we applied the same sequencing and analysis methods to the cell line obtained from the reference sample NA12878 (Methods). Through the application of the GATK‐SV pipeline, 10 624 SVs were detected. A comparison of the detected SVs with the gold standard truth set of NA12878 [45] revealed a precision of 79.5% for deletions, 64.2% for duplications, 69.4% for insertions, and 75% for inversions. Because NA12878 represents a different ancestry background from our study population, we further benchmarked the SV calling performance using a high‐confidence Chinese genome from The Quartet Project. This ancestry‐matched evaluation yielded precision estimates of 84.4%, 65.3%, and 78.2% for deletions, duplications, and insertions, respectively. Compared with NA12878, insertion precision showed the largest improvement, suggesting that population‐matched benchmarking may better capture SV detection performance in the target population. Additionally, the impact of sequencing depth on SV detection was assessed, as shown in Figure S4. Overall, a higher sequencing depth was correlated with an increased number of SVs, with a gradual rise in SV counts from 20× to 50×. However, this trend declined beyond 50×, potentially owing to the limited number of samples used.

Across all population‐detected SVs, 71.55% were rare, with minor allele frequencies (MAF) below 1%. Most SVs exhibited lengths distributed within the range of 50–100 bp (61.02%) and 100–500 bp (15.77%; Figure 1c). The expected peaks corresponding to the insertion of Alu, SVA, and LINE1 mobile elements were identified at approximately 300 bp, 2 kb, and 6 kb, respectively (Figure 1d). These SV patterns were consistent with those reported in previous studies.

Regarding the functional prediction of SVs, a significant proportion of SVs were located in intergenic (57.32%) or intronic regions (36.62%; Figure 1e). Only 6.06% of SVs breakpoints intersected with functional elements, including exons (n = 3381), predicted loss‐of‐function (pLoF; n = 1895), copy gain of entire genes (CG; n = 1170), intragenic exonic duplication (IED; n = 1308), and promoters (n = 655). On average, each genome harbored a mean of 173.96 SVs that altered the functions of 213 genes (121.37 pLoF affected 122.7 genes, 26.09 CG affected 65.4 genes, and 25.69 IED affected 25.1 genes; Figure 1f), indicating that approximately 1.1% of human protein‐coding genes per individual could experience functional abnormalities due to SVs.

To ascertain whether the SVs identified in this study had been previously documented, we compared them with five publicly available SV databases: gnomAD [42], Audano et al. 2019 [46], the Human Genome Structural Variation Consortium (HGSVC2) [47], Wu et al. [38], and dbVar [48] (accessed on September 30, 2023). Initially, we ensured that the SV coordinates in these databases were converted to GRCh38 to align with our datasets. Because insertion events exhibit greater breakpoint uncertainty than other SV classes, overlap criteria were defined according to SV type. For non‐insertion SVs, variants were considered overlapping when ≥50% reciprocal overlap was observed. For insertion variants, two SVs were considered matched when their breakpoint distance was ≤20 bp, representing a conservative criterion for assessing overlap between independently identified insertion events compared with some previous studies [49, 50, 51]. Using these criteria, 77 385 (55.7%) SVs showed overlap with at least one previously reported SV resource, whereas 61 474 (44.3%) SVs were not represented in the compared databases (Figure 1g). Among the previously reported SVs, the highest number of shared variants was observed with dbVar (75 292; 54.22%), followed by gnomAD (71 065; 51.18%), Wu et al. (48 024; 34.58%), Audano et al. (19 223; 13.85%), and HGSVC2 (16 303; 11.74%). The relatively high overlap with Wu et al., a long‐read WGS‐based SV resource from 405 Chinese individuals, likely reflects the shared ancestry and population background between the two datasets. Notably, 94.7% (58 220/61 474) of these unrepresented SVs were insertions and showed enrichment in intergenic and intronic regions (Figure 1h). Consistent with their genomic distribution, these variants showed limited overlap with functional elements, affecting an average of 20.61 SV‐altered genes per genome, including 6.95 pLoF, 2.24 CG, and 11.41 IED (Figure 1i). To assess the robustness of insertion novelty estimation, we further evaluated insertion overlap using relaxed breakpoint distance thresholds. Increasing the breakpoint distance threshold from 20 bp to 50, 100, 200, 500, and 1000 bp progressively increased the number of reported insertions (Figure S5). These results suggested that the high proportion of unreported SVs was primarily driven by the limited representation and benchmarking challenges of insertion events in existing population SV catalogs, rather than solely reflecting limitations in SV detection accuracy.

### 2.2 Associations of SV Burden and Common SVs With the Gut Microbiome

After quality control during SV calling, 1406 of the 1839 samples had available fecal shotgun metagenomic sequencing data. We first assessed whether the overall burden of host SVs was associated with gut microbial diversity. Using a generalized linear regression model adjusted for demographic, diet, and lifestyle covariates (Methods), we observed a modest positive correlation between total SV burden and α‐diversity indices (Shannon index: β = 0.08, *p* = 0.011; Simpson index: β = 0.06, *p* = 0.012; Figure S6). We then calculated the associations of 22 519 common SVs (MAF ≥ 1%) with α‐diversity indices and identified no significant associations after multiple test correction (*p* < 2.22 × 10^−6^ = 0.05/22,519; Figure S7), consistent with previous microbiome GWAS studies showing limited genetic contribution to global diversity metrics but for specific microbial taxa/pathways (Table S2).

We next performed mbGWAS of 22 519 common SVs and 616 microbial features comprising 517 gut microbial taxa and 99 gut metabolic modules (GMMs) present in over 10% of the individuals (Table S3). A total of 30 SVs (DEL = 16, INS = 7, DUP = 6, INV = 1) were identified to be associated with gut microbial features at genome‐wide significance (*p* < 2.22 × 10^−6^; Figure 2a and Table S4). Association‐level quality assessment of these significant SV loci, including MAF, HWE statistics, supporting evidence from SV calling, and overlap with public SV resources, supported the reliability of these significant SV loci (Table S5). Further, we evaluated potential statistical inflation in mbGWAS analyses. The genomic inflation factor (λ) ranged from 0.84 to 1.33, with a median value of 0.98 (Figure 2b), indicating no obvious evidence of inflation, except for some specific mbGWAS tests.

**FIGURE 2:**
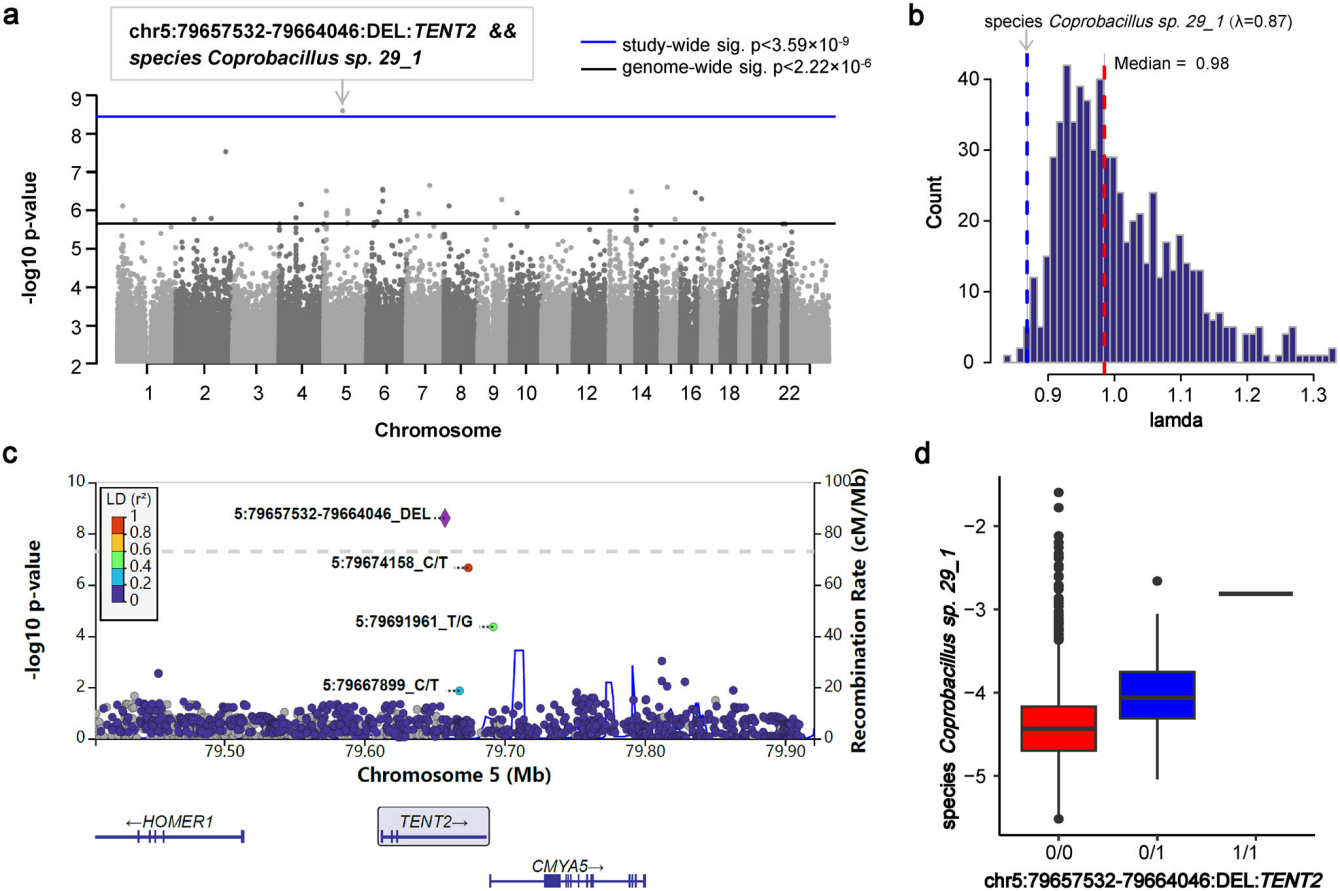
Genome‐wide associations of SVs with the gut microbiome identified by mbGWAS. (a) Manhattan plot representing the GWAS results (619 microbial features; 22,519 common high‐quality SVs with MAF ≥ 1%). The y‐axis indicates the log10‐transformed *p*‐values, while the x‐axis indicates the genomic positions of the SVs. The study‐wide significance (*p* < 3.59 × 10^−9^) and genome‐wide significance (*p* < 2.2 × 10^−6^) are indicated by horizontal lines colored blue and black, respectively. The annotations highlight the study‐wide association of the deletion variant in the intronic region of the *TENT2* gene and the species *Coprobacillus sp. 29_1*. (b)The distribution of genomic inflation factor (λ) for 616 mbGWAS tests, ranging from 0.84 to 1.33, with a median value of 0.98. The λ of GWAS for species *Coprobacillus sp. 29_1* is 0.87. (c) The regional plot showing mbGWAS results of the *TENT2* deletion (purple diamond) and its nearby SNPs associated with the species *Coprobacillus sp. 29_1*. (d) The log‐transformed relative abundances of *Coprobacillus sp. 29_1* in carriers of the *TENT*2 deletion and non‐carriers.

Only one association achieved the study‐wide significance level (*p* < 3.60 × 10^−9^, adjusting for 22,519 SVs and 616 microbial features). To evaluate the contribution of host SVs to gut microbiome traits, we estimated the proportion of phenotypic variance explained by significant SVs using the GCTA‐REML approach. Among the microbial features with significant SV associations, the variance explained by associated SVs ranged from 1.2% to 3.2% (Table S6), with the highest estimate observed for genus *Turicibacter* (h^2^ = 3.19%, SE = 3.22%), followed by species *Coprobacillus sp. 29_1* (h^2^ = 2.59%, SE = 3.68%) and MF0063 (h^2^ = 2.22%, SE = 3.17%). These estimates suggest modest SV contributions but should be interpreted cautiously given the relatively large standard errors for some traits.

To further benchmark the contribution of SVs against measured environmental factors at the community level, we performed Bray–Curtis β‐diversity variance‐partitioning analyses. After adjustment for age, sex, genetic PCs, and 12 diet/lifestyle variables, the 30 microbiome‐associated SVs explained an additional 3.32% of species‐level and 3.75% of genus‐level community variation (adjusted R^2^ = 1.26% and 1.71%, respectively; permutation *p* = 0.001 for both; Table S7). In comparison, the 12 measured diet/lifestyle variables explained 1.85% and 2.11% of species‐ and genus‐level variation beyond age, sex, and genetic PCs (adjusted R^2^ = 1.01% and 1.27%, respectively). These analyses demonstrate that the prioritized SVs capture a modest but measurable component of microbiome variation that remains detectable after accounting for measured lifestyle‐related factors.

The most significant association was observed for a 6.5 kb deletion (chr5:79657532‐79664046) within the intron region of the gene *TENT2*, which was positively correlated with the relative abundance of the species *Coprobacillus sp. 29_1* (β = 0.158, *p* = 2.50 × 10^−9^; Figure 2c). Carriers of this SV exhibited significantly higher mean relative abundances of *Coprobacillus sp. 29_1* (homozygous DEL: 0.15%; heterozygous DEL: 0.024%) than the non‐carriers (0.015%; Figure 2d). This GWAS test exhibited no inflation, with a λ value of 0.87. The identified SV had an MAF of 0.014 in this cohort and was specific to East Asian (MAF = 0.028 for ASN; MAF = 0 for other populations) according to the 1000 Genomes database [52]. Further analysis revealed that this SV was correlated with concentrations of glycine (*p* = 0.015), ethanol (*p* = 0.022), monocytes (*p* = 0.027), and albumin (*p* = 0.028) by investigating its associations with blood parameters in this cohort (Figure S8). Interestingly, *Coprobacillus sp. 29_1* associated SV also showed a correlation with monocyte concentration, suggesting potential interactions among the three. By calculating the pairwise linkage disequilibrium (LD) between this SV and its neighboring SNPs, we found that this SV was in strong LD with the SNP chr5:79674158_C/T (r^2^ = 0.973; Figure 2c), which also resided in the intronic region of *TENT2* and showed a significant association with *Coprobacillus sp. 29_1* (β = 0.143, *p* = 2.18 × 10^−7^). Consistent with SV, this SNP had a MAF of 0.013 in this cohort and 0.027 in the ASN population but was absent in other populations from the 1000 Genomes database. Due to the strong LD between the SV and SNP, the association with *Coprobacillus sp. 29_1* became insignificant after conditioning on either of them.

In addition to the study‐wide significant signal, 22 SVs were associated with microbial taxa at genome‐wide significance (Table S4). Among them, three SVs lied in genes that were extremely intolerant to pLoF variants with pLI ≥ 0.99, as annotated by the AnnotSV tool. Specifically, a 61‐bp duplication within the *CDH8* gene was negatively associated with the abundance of *Streptococcus sanguinis* (β = ‐0.137, *p* = 3.37 × 10^−7^), a predominant bacterium in the human oral cavity known to be linked to the development of infective endocarditis [53]. Similarly, a 108 bp duplication in the *ANKRD11* gene was negatively associated with the abundance of *Turicibacter* (β = ‐0.133, *p* = 4.91 × 10^−7^). The SV overlapped with pathogenic loss of *ANKRD11*, which caused KBG syndrome, a disease characterized by intellectual disability, skeletal malformations, and macrodontia [54]. *Turicibacter* has been reported to be a highly heritable taxon in both human and mouse genetic studies [12, 55, 56], supporting the association between SV and *Turicibacter* abundance found in this study. Conversely, a 751 bp deletion in the *GRID2* gene was positively associated with the presence of *Eubacterium cellulosolvens* (β = 0.755, *p* = 6.85 × 10^−7^), an anaerobic cellulolytic bacterium in the gut. The *GRID2* gene encodes the glutamate receptor channel delta‐2 subunit, and its deletion leads to a recessive syndrome of cerebellar ataxia and tonic upgaze in humans [57].

In addition to the three SVs within disease‐associated genes, several SVs were found to exhibit significant effects on potential probiotics that could contribute to host health. For example, a duplication event in the *LOC107985978* gene on chromosome 7 (SV length: 68 bp) was positively associated with the abundance of *Roseburia intestinalis* (β = 0.139, *p* = 2.20 × 10^−7^), a promising probiotic known for its anti‐inflammatory properties by producing butyrate [58, 59]. An insertion on chromosome 15 involving an ALU element (SV length: 262 bp) demonstrated a significantly negative correlation with the emerging probiotic *Parabacteroides distasonis* [60] (β = ‐0.137, *p* = 2.51 × 10^−7^).

In addition to microbial taxa, seven associations were identified for the GMMs, also known as microbial pathways. For instance, the glyoxylate bypass pathway (MF0063) was negatively associated with an insertion on chromosome 2 (SV length: 111 bp, β = ‐0.147, *p* = 2.92 × 10^−8^). The pyruvate dehydrogenase complex (MF0072) exhibited a negative association with a deletion on chromosome 13 (SV length: 234 bp, β = ‐0.132, *p* = 3.26 × 10^−7^). The pectin degradation II pathway (MF0004) exhibited a negative association with a deletion on chromosome 9 (SV length: 82 bp, β = ‐0.131, *p* = 5.19× 10^−7^). Notably, all the SVs associated with the microbiome were located in the intronic and intergenic noncoding regions, except for an inversion of 4.78Mb spanning 14 genes, consistent with previous findings in the SNP‐based mbGWAS [61].

### 2.3 Conditional Analyses and Functional Annotations of Microbiome‐Associated SVs

To evaluate whether the identified microbiome‐associated SV associations were explained by nearby SNP variation, we calculated pairwise LD between SVs and neighboring SNPs within the ±500 kb flanking regions. We found that eight of the 30 microbiome‐associated SVs exhibited at least moderate LD (r^2^ ≥ 0.5) with one SNP or INDEL associated with the same microbial feature (Figure 3a and Tables S4 and S8). We subsequently performed conditional analyses by adjusting for all independent (r^2^ < 0.1) and significant (*p* < 0.01) SNP associations within each SV locus.

**FIGURE 3:**
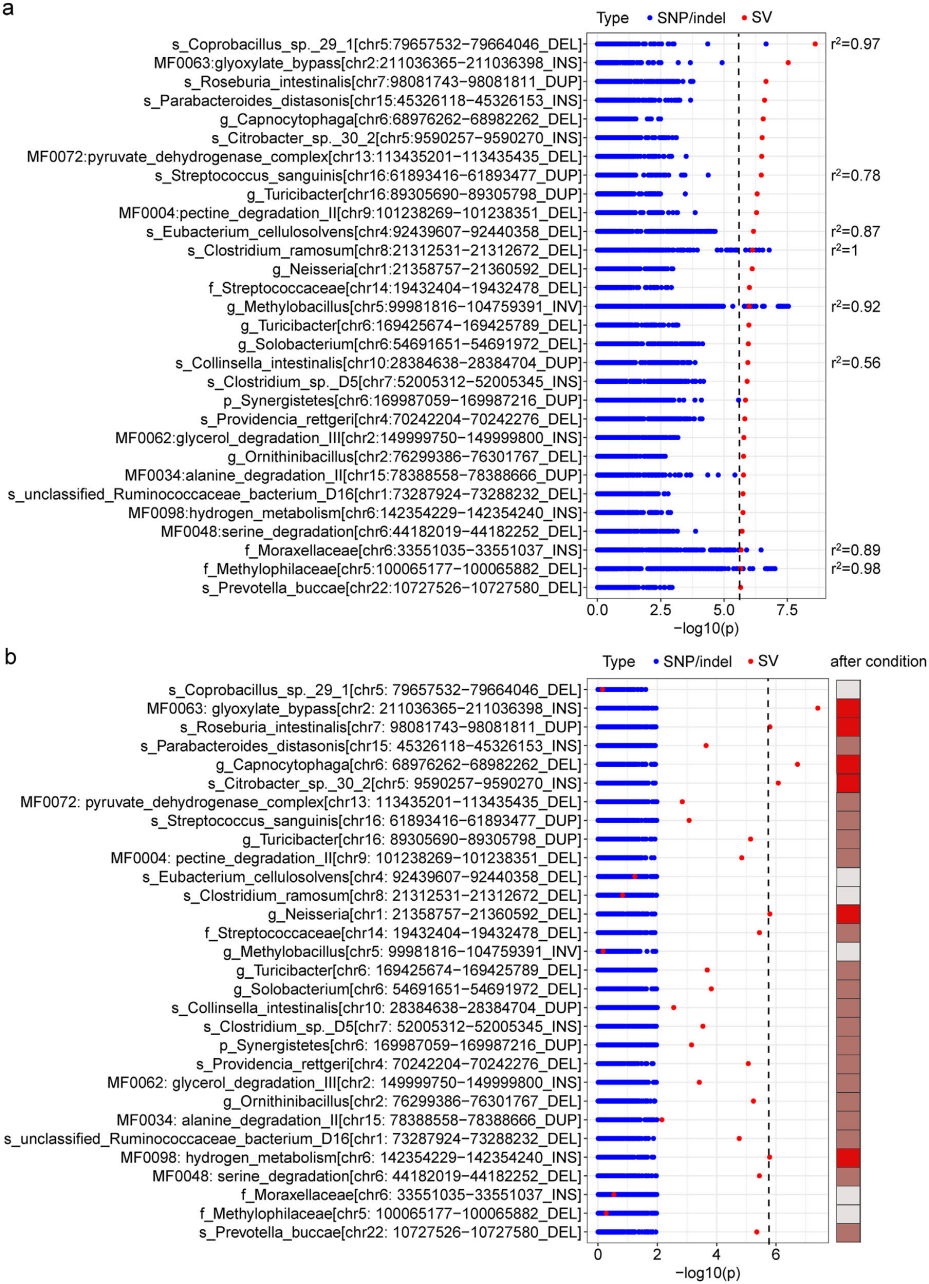
The raw and conditional GWAS results for significant SVs and their nearby SNPs/INDELs with the gut microbiome. (a) The raw associations of SVs and their nearby SNPs/INDELs (flanking 500 kb of the SV) with the gut microbiome. (b)The associations of SVs and SNPs/INDELs with the gut microbiome after conditioning on all the nearby independent SNPs/INDELs with *p*<0.01. The bar on the right indicates the significance of SV association after conditional analysis, including no longer significant (colored in grey), not reaching genome‐wide significance (colored in dark red), and retaining genome‐wide significance (colored in red). The x‐axis indicates the log10‐transformed p‐values. The y‐axis indicates the associations of the SVs and nearby SNPs/INDELs with the gut microbiome (N = 30). The red dots represent the *p*‐values associated with SVs, and the blue dots represent the *p*‐values associated with SNPs/INDELs. The vertical dashed lines represent the genome‐wide *p*‐value of 2.22 × 10^−6^.

After conditioning, six SV–microbiome associations were attenuated to *p* > 0.01, 18 remained associated at p ≤ 0.01 but no longer reached genome‐wide significance, and six retained genome‐wide significance (*p* < 2.22×10^−6^; Figure 3b). Thus, only a subset of SV associations retained genome‐wide significance after accounting for local SNP signals, indicating that these SV associations were not fully explained by the tested local SNP variation. Two retained SV signals were potentially linked to the probiotics—*Roseburia intestinalis* and *Parabacteroides distasonis*. To further assess whether microbiome‐associated SVs capture additional information beyond SNP associations at the community level, we performed Bray–Curtis β‐diversity variance‐partitioning analyses in individuals with complete microbiome and genetic data. After adjustment for age, sex, and genetic principal components, the significant SV block explained additional microbiome variation after accounting for prioritized SNP associations, contributing 1.05% and 1.39% adjusted ΔR^2^ at the species and genus levels, respectively (permutation *p* = 0.002 and 0.007; Table S7). Although the significant SNP block explained a larger proportion of microbiome variation overall, these results indicate that SVs capture complementary components of host genetic variation that are not completely represented by the tested SNP signals.

AnnotSV [62, 63, 64] was employed to annotate the 30 microbiome‐associated SVs, identifying 24 genes that harbored these SVs. Subsequently, gene enrichment analysis was conducted for these 24 genes. Gene Ontology (GO) enrichment analysis revealed 10 significantly enriched GO terms (*p*.adjust < 0.05), primarily involving the structure and function of neuronal synapses, encompassing the synaptic membrane, synaptic cleft, sodium‐independent organic anion transport, sodium‐independent organic anion transmembrane transporter activity, postsynaptic specialization membrane, postsynaptic density membrane, glutamatergic synapse, and excitatory synapse (Figure S9a and Table S9). Additionally, Kyoto Encyclopedia of Genes and Genomes (KEGG) enrichment analysis indicated significant enrichment of these genes in four pathways: nicotinate and nicotinamide metabolism, long‐term depression, the peroxisome, and the phosphatidylinositol signaling system (Figure S9b and Table S10). These findings suggested a potential role of microbiome‐associated SVs in neurotransmission, ion transport, and cell signaling pathways.

### 2.4 Associations of Rare SVs and the Gut Microbiome

To further explore the relationship between rare SVs (MAF < 0.01) and the gut microbiome, we performed a gene‐based sequence kernel association test (SKAT) analysis of overall diversity and microbial abundances. With a *p*‐value threshold of 8 × 10^−6^ by adjusting for the detected 5861 genes, we did not observe a significant association between overall diversity and rare SV burden. However, 56 gene loci were associated with microbial features (Table S11). Two associations reached study‐wide significance (*p* < 1.38 × 10^−9^ by adjusting for 5861 genes and 616 microbial features). The strongest signal was observed for the Janus kinase and microtubule interacting protein 2 (*JAKMIP2*) gene (harboring four rare SVs) associated with the abundance of MF0103: mucin degradation (*p* = 8.28 × 10^−10^). The *JAKMIP2* gene has been reported as a potential risk factor for Graves' disease in the Chinese Han population [65], and its nearby long noncoding RNA, *JAKMIP2‐AS1*, promoted the growth of colorectal cancer and indicated poor prognosis [66]. Accordingly, mucin degradation has been linked to intestinal inflammation and tumor incidence [67]. The second strongest signal lied in the gene *SLC9A3* (encoding NHE3: sodium‐hydrogen antiporter/exchanger 3; harboring seven rare SVs) associated with the abundance of MF0021: xylose degradation (*p* = 1.28 × 10^−9^). This gene had altered expression in the colonic mucosa of adult IBD patients [68], showed strong epigenetic interaction with the microbiome [69], and *SLC9A3* knockout mice developed colitis [70].

In addition to these pathways, some genes were associated with specific microbial taxa. For example, several genes, including *INTS9*, *HLA‐DQA1*, and *MTMR3*, were associated with the species *C. intestinalis* from the genus *Collinsella*, which was linked to serum cholesterol level [71] and liver‐related disease [72]. The potassium voltage‐gated channel subfamily B member 1 (*KCNB1*) SV burden was correlated with the abundance of *Streptococcus salivarius* (p = 3.88 × 10^−7^), *S. vestibularis* (*p* = 6.06 × 10^−7^) and *S. sp.C150* (*p* = 4.23 × 10^−6^) (Table S11). A mouse study demonstrated that *S. salivarius* modulates sucrose metabolism via exopolysaccharide (EPS)‐dependent short‐chain fatty acid production [73], while *KCNB1* variants impaired pancreatic β‐cell function and increased the risk of type 2 diabetes (T2D) [74]. This dual regulation—microbial EPS‐SCFA axis and host ion channel activity—suggests a synergistic mechanism whereby *KCNB1* SVs exacerbate T2D risk by disrupting microbial carbohydrate processing and pancreatic glucose sensing. These “host genes‐gut microbiome” links need to be further investigated in the future.

### 2.5 Associations of SVs With Blood Metabolites

Next, we conducted a GWAS for 22 519 common SVs and 121 blood metabolic traits. A total of 48 associations involving 33 SVs (DEL = 11, INS = 14, DUP = 8) reached genome‐wide significance (*p* < 2.22 × 10^−6^ = 0.05/22519; Figure 4a and Table S12). Among these, 17 associations reached study‐wide significance (*p* < 1.835 × 10^−8^, adjusting for 22 519 SVs and 121 blood metabolite traits). The genomic inflation factor λ was calculated, with a median value of 1 (ranging from 0.80 to 1.33; Figure S10). Associated SVs explained measurable proportions of trait variance, ranging from 0.94% to 31.5% across significant blood traits (Table S13), with the highest estimated contributions observed for mean corpuscular volume (h^2^ = 31.52%, SE = 21.68%), followed by mean corpuscular hemoglobin (h^2^ = 27.41%, SE = 20.00%) and mean corpuscular hemoglobin concentration (h^2^ = 9.79%, SE = 12.58%).

**FIGURE 4:**
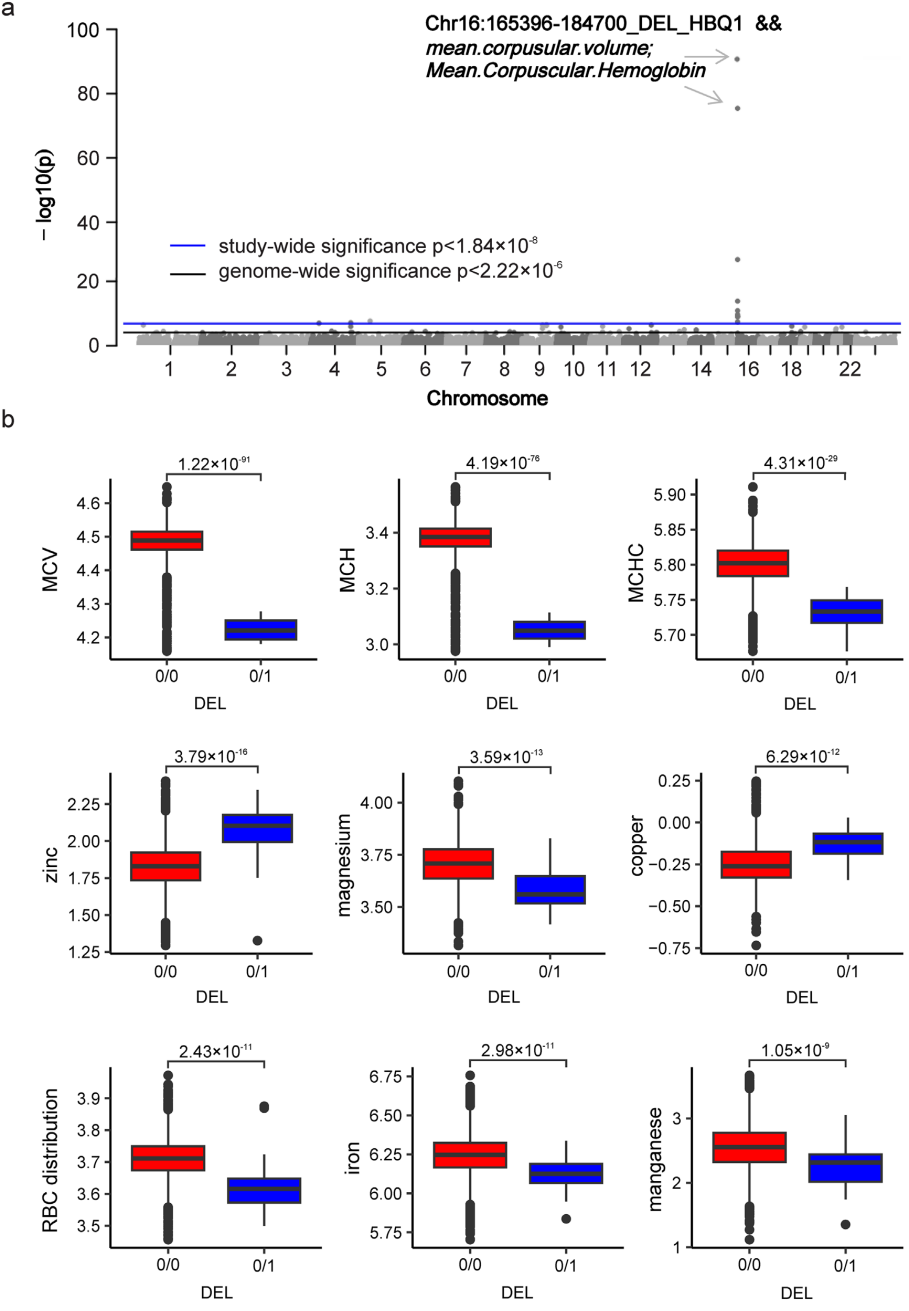
Genome‐wide associations of SVs with blood metabolites. (a) Manhattan plot depicting the GWAS results (121 blood metabolite traits; 22,519 common high‐quality SVs with MAF ≥ 1%). The study‐wide significance (*p* < 1.84 × 10^−8^) and genome‐wide significance (*p* < 2.22 × 10^−6^) are indicated by horizontal lines colored blue and black, respectively. The annotations show the DEL variant on chromosome 16 (chr16: 165396–184700), corresponding to the *HBA1*, *HBA2*, *HBM*, and *HBQ1* genes. (b) Boxplots of nine blood metabolite levels associated with the *HBA1/HBA2/HBM/HBQ1* DEL that passed study‐wide significance, comparing the metabolite levels between DEL carriers (“0/1”: individuals with DEL) and non‐carriers (“0/0”: individuals without DEL). MCV, mean corpuscular volume; MCH, mean corpuscular hemoglobin; MCHC, mean corpuscular hemoglobin Concentration; RBC distribution, red blood cell distribution.

The strongest association signal was observed for a deletion on chromosome 16 (chr16:165396‐184700; SV length: 19,304 bp) spanning the *HBA1*, *HBA2*, *HBM*, and *HBQ1* genes, known as the widely reported Southeast Asian deletion (–^SEA^), which is predominantly distributed and associated with anemia in southern China and Southeast Asia [75, 76]. In the Chinese population in this study, the MAF of the SEA deletion was 0.01. Consistently, the deletion was mainly detected in East Asian (ASN, MAF = 0.019) and South Asian (SAN, MAF = 0.013) populations in the 1000 Genomes dataset. The deletion showed remarkably significant associations with nine blood metabolic traits, particularly with mean corpuscular volume (MCV, β = ‐0.570, *p* = 1.22 × 10^−91^), mean corpuscular hemoglobin (MCH, β = ‐0.522, *p* = 4.19 × 10^−76^), mean corpuscular hemoglobin concentration (MCHC, β = ‐0.319, *p* = 4.31 × 10^−29^), and red blood cell distribution (RBC, β = ‐0.198, *p* = 2.43 × 10^−11^; Figure 4 and Table S12). Beyond its established correlations with blood cell parameters, the deletion was also associated with blood microelement levels. Carriers of the deletion exhibited elevated zinc and copper concentrations, whereas magnesium, iron, and manganese levels were decreased compared to non‐carriers (Figure 4b). In addition to the 16:165396‐184700 deletion, a 114‐bp duplication on chromosome 9 (chr9:97255555‐97255669) within the *SUGT1P4*‐*STRA6LP*‐*CCDC180* gene was also associated with MCV (β = ‐0.174, *p* = 7.89 × 10^−9^) and MCH (β = ‐0.174, *p* = 7.89 × 10^−9^) levels. This DUP had an MAF of 0.0125 in this study, which has not been previously reported.

Among the 48 genome‐wide associations, cystine, previously reported as the most heritable metabolite in Chinese [14], exhibited the highest number of associated SVs, with 16 SVs linked to it (Figure 5a). Three SVs reached study‐wide significance, including a 9.2 kb deletion on chromosome 5 (5:46048650‐46057870; β = ‐0.163, *p* = 6.47 × 10^−10^) and two insertions on chromosome 4 (β = ‐0.161, *p* = 2.83 × 10^−9^) and 12 (β = ‐0.161, *p* = 2.83 × 10^−9^), respectively (Table S12).

**FIGURE 5:**
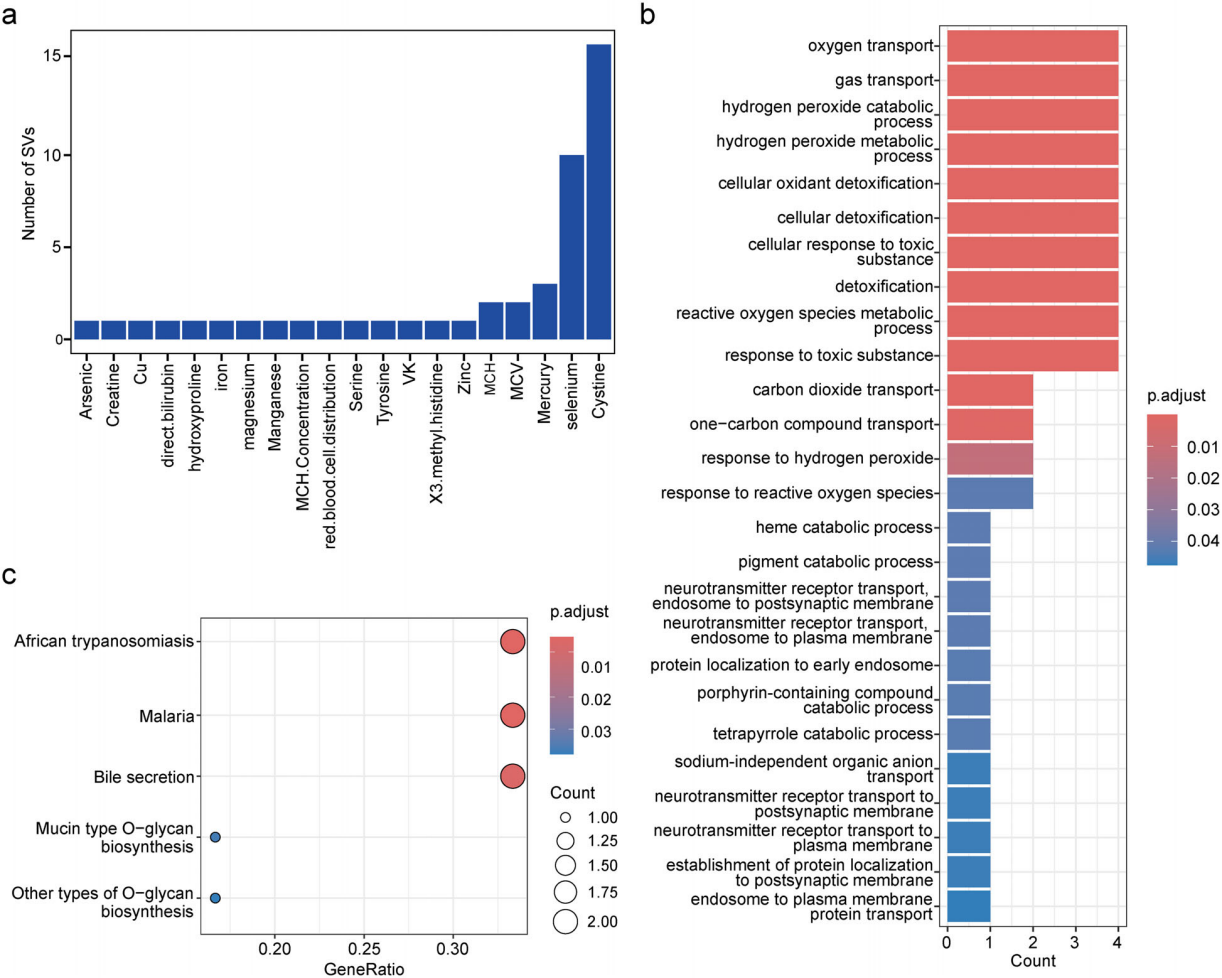
The enrichment analysis of significantly associated SVs in blood metabolites. (a) The distribution shows the number of SVs significantly associated with blood metabolites (N = 48). (b and c) GO (b) and KEGG enrichment analysis (c) for genes harboring SVs significantly associated with blood metabolites. The bar plot (b) only displays the biological process (BP) results from the GO analysis terms.

The results of GWAS and LD analysis revealed that three of the 33 SVs appeared to have weaker associations with their respective metabolites than neighboring SNPs and could be tagged by neighboring SNPs with r^2^> 0.5 (Figure S11). Conditional analysis further confirmed that the associations of the three SVs with metabolites were attenuated even not significant after adjusting for the neighboring SNPs. For example, the *HBA1*/*HBA2*/*HBM*/*HBQ1* deletion was in moderate LD with the lead SNP chr16:199621 across the nine metabolic traits (r^2^ = 0.76). After conditioning on the SNP, the association of the deletion with these traits was notably weakened, although it remained statistically significant for six of them (*p*<0.01; Table S12), highlighting the significance of this SV in influencing host metabolic processes that are not completely accounted for by nearby SNPs. Furthermore, a 64 bp insertion (chr19:15866773‐15866783) associated with vitamin K concentration was in strong LD with its tagging SNP (r^2^ = 0.94), while a ∼6 kb insertion (chr12:20861159‐20861190) associated with the direct bilirubin level was in moderate LD with its tagging SNP (r^2^ = 0.76). The associations between the two insertions and metabolites were not significant after adjusting for the neighboring SNPs. Apart from these three SVs, 20 of the remaining 30 SVs continued to exhibit significant links with their respective metabolites after conditional analysis, underscoring the independent regulatory roles of these SVs in blood metabolic traits (Tables S12 and S14).

Gene‐based analysis of rare SVs identified 22 unique genes associated with blood metabolic metabolites and further confirmed the most correlations with cystine (Table S15). SV burdens involving six genes, including *CLN3*, *ELF2*, *WDFY4*, *RBPMS*, *IL6ST*, and *SORCS3*, were linked to cystine levels. The most significant and the only one passing multiple test correction (*p* < 7.6 × 10^−8^ by adjusting for 5861 genes and 121 metabolites) was for *the CLN3* gene, which encodes a protein that is involved in lysosomal function and is prenylated most likely at cysteine 435 [77]. The other candidate associations were listed in Table S15.

GO enrichment analysis of the gene sets harboring significant SVs identified 26 BP, 16 CC, and 30 MF terms (Figure 5b and Table S16). The enriched gene sets predominantly featured biological process pathways encompassing gas transport, detoxification and redox, protein and receptor transport, metabolism, and degradation, indicating that SVs associated with blood metabolites play a pivotal role in these biological processes. Additionally, KEGG enrichment analysis revealed a notable enrichment of the gene sets in pathways associated with infectious diseases (e.g., African trypanosomiasis, malaria), bile secretion, and glycosylation‐related metabolic pathways (Figure 5c and Table S17), implying that SVs located near these genes played crucial roles in these metabolic pathways.

### 2.6 Replication of Previously Reported Metabolites‐Associated SVs

In the present study, we investigated previously reported metabolites‐associated SVs to determine their replication. Out of a total of 58 autosomal SVs identified in previous studies [36, 37, 38], 33 were detected in our samples, with 16 showing associations with metabolites (*p*< 0.05; Tables S18–S20). Notably, following a stringent *Bonferroni* correction threshold of 0.00152 (0.05/33), three SVs remained statistically significant. The most robust signal was observed for the *HBA1*/*HBA2*/*HBM*/*HBQ1* (16:165396‐184701) SV, which was associated with MCV/MCH, as confirmed by our study, the NHLBI TOPMed program, and the study by Wu et al. Additionally, the SV within the growth hormone receptor (GHR) gene, previously linked to the small round epithelial cell (SRC) count in the study by Wu et al., was found to be associated with multiple metabolites, including cystine, aspartic acid, and glutamic acid, in our study. Moreover, the duplication involving the T cell receptor alpha variable (TRAV) genes, known to be associated with C‐reactive protein (CRP) levels in the study by Chen et al., was also correlated with valine levels in our study. These replicated associations across different studies underscore the importance of some specific SVs in human metabolism, emphasizing the need for further validation in larger populations to confirm additional potential associations and to advance our understanding in this domain.

### 2.7 Potential Causal Links Between Microbiome and Metabolites Inferred by Host SVs

Furthermore, we investigated the extent to which microbiome‐ and metabolite‐associated SV loci were shared, aiming to characterize common genetic architecture underlying host–microbiome–metabolite interactions. Among the 30 microbiome‐associated SVs, 29 were also nominally associated with at least one blood metabolic trait (*p* < 0.05), whereas all 33 metabolite‐associated SVs showed nominal associations with at least one microbial feature (Figure 6a,b and Tables S21–S22). These shared associations suggest coordinated genetic influences of host SV variation on gut microbial and metabolic traits, but do not by themselves establish directional relationships.

**FIGURE 6:**
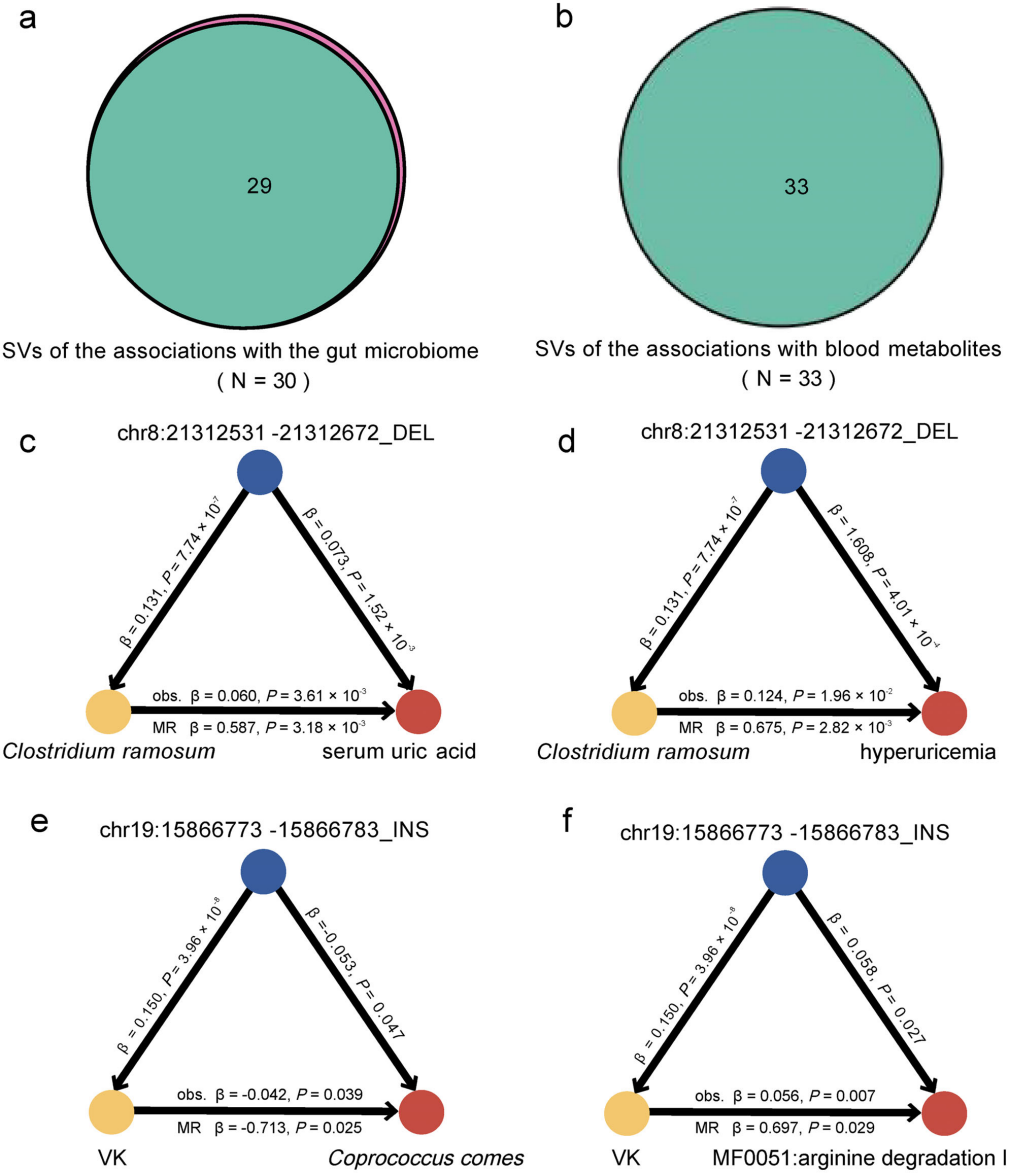
Potential causal links between the gut microbiome and blood metabolites supported by SVs. (a) Venn diagrams showing the number of overlapping SVs significantly associated with the gut microbiome (*p* < 2.2 × 10^−6^) that also linked to blood metabolites (*p* < 0.05). (b) Venn diagrams showing the number of overlapping SVs significantly associated with the blood metabolites (*p* < 2.2 × 10^−6^) that also linked to the gut microbiome (*p* < 0.05). (c) The deletion on chromosome 8 (SV length: 141 bp) was significantly associated with both Clostridium ramosum (β = 0.131, *p* = 7.47 × 10^−7^) and serum uric acid (β = 0.073, *p* = 1.52 × 10^−3^). A significant observational correlation and an MR‐supported positive association of Clostridium ramosum with serum uric acid. (d) The deletion on chromosome 8 (SV length: 141 bp) was significantly associated with both Clostridium ramosum (β = 0.131, *p* = 7.47 × 10^−7^) and hyperuricemia (β = 1.608, *p* = 4.01 × 10^−4^). Observational correlation and MR analyses consistently supported a positive association of *Clostridium ramosum* with hyperuricemia. (e) An MR‐supported negative association of vitamin K with *Coprococcus comes*, instrumented by the insertion of the *CYP4F2/CYP4F11/OR10H1/OR10H5* locus (chr19:15866773 ‐15866783). (f) An MR‐supported positive association of vitamin K with microbial pathway MF0051:arginine degradation I, instrumented by the insertion of the *CYP4F2/CYP4F11/OR10H1/OR10H5* locus (chr19:15866773 ‐15866783). Obs., Observational Correlation; MR, Mendelian Randomization. MCHC, Mean Corpuscular Hemoglobin Concentration.

We next directly assessed phenotypic correlations between microbial features and metabolic traits. Applying stringent criteria (associated SV with *p* < 2.22 × 10^−6^ for one phenotype, *p* < 0.05 for the other, and *p* < 0.05 for their pairwise correlation), we identified 21 significant “microbial feature–metabolic trait” pairs (Table S21). For example, the 141‐bp deletion on chromosome 8 associated with *Clostridium ramosum* (β = 0.131, *p* = 7.47 × 10^−7^) in the mbGWAS was also correlated with the serum uric acid level (β = 0.073, *p* = 1.52 × 10^−3^) and hyperuricemia (β = 1.608, *p* = 4.01 × 10^−4^; Table S23). Consistently, *Clostridium ramosum* showed significant positive correlations with both serum uric acid (β = 0.060, *p* = 3.61 × 10^−3^) and hyperuricemia (β = 0.124, *p* = 0.02; Figure 6c,d). Similarly, a chromosome 5 insertion associated with *Citrobacter* abundance was also linked to circulating 1‐methylhistidine levels, showing concordant associations between microbial and metabolic traits.

Conversely, 325 “metabolic trait—microbial feature” pairs were identified using analogous criteria (Table S22). For example, the *HBA1*/*HBA2*/*HBM*/*HBQ1* deletion associated with nine blood metabolites such as MCHC and zinc was also correlated with the microbial pathway MF0064 (triacylglycerol degradation) (β = ‐0.082, *p* = 6.47 × 10^−10^). Significant correlations were observed between MCHC and MF0064 (β = 0.075, *p* = 2.85 × 10^−3^) and between zinc and MF0064 (β = ‐0.039, *p* = 4.95 × 10^−2^). These results suggest coordinated associations among host SVs, gut microbes, and metabolites.

To explore potential directional relationships between microbial features and metabolic traits, we performed SV‐based Mendelian randomization (MR) analyses. All instrumental variables were LD‐pruned and showed sufficient instrument strength, with F‐statistics exceeding the conventional threshold of 10 (mean F‐statistic: 27.39 for microbiome and 67.20 for metabolite instruments; Figure S12). MR analysis identified 29 MR‐supported associations involving 25 microbial traits and 14 metabolic traits (Table S24). Among these, observational correlation and MR estimates consistently supported positive effects of *Clostridium ramosum* on serum uric acid level and hyperuricemia risk (Figure 6c,d), and of *Citrobacter* on increased 1‐methylhistidine level. Blood MCHC, zinc, and manganese showed positive effects on the triacylglycerol degradation pathway. Tyrosine showed MR‐supported associations with abundances of diverse gut microbiota, including *Salmonella enterica*, *Bacteroides intestinalis*, MF0052 (arginine degradation II), Gammaproteobacteria, *Providencia rettgeri*, MF0093 (propionate production I), and Enterobacteriaceae. Vitamin K was genetically associated with the reduced abundance of *Coprococcus comes* but increased abundance of the arginine degradation I pathway (Figure 6e,f).

We further performed an additional summary‐statistics‐based MR analysis to evaluate the robustness of these associations. Because both exposure and outcome summary statistics were derived from the same cohort, this analysis was considered a sensitivity assessment rather than independent validation. The analysis identified 202 significant MR‐supported associations involving 29 metabolic and 128 microbial traits (Table S25). All associations identified in the primary MR analysis were directionally consistent in this sensitivity analysis, including the positive association of *Clostridium ramosum* with serum uric acid (β = 0.554, *p* = 1.48 × 10^−^^3^) and *Citrobacter* with 1‐methylhistidine (β = 0.590, *p* = 4.16 × 10^−^^3^), as well as the negative association of vitamin K with *Coprococcus comes* (β = ‐0.353, *p* = 0.047).

### 2.8 In Vitro Experimental Validation

To provide experimental support for selected metabolite–microbe relationships identified in the MR analysis, we examined three representative pairs involving different predicted directions of effect. These pairs were selected based on their MR‐predicted associations, availability of representative cultured strains, and feasibility of controlled in vitro testing.

We first evaluated the previously identified negative association between vitamin K (VK) and *Coprococcus comes*. Because vitamin K comprises multiple vitamers, including mainly vitamin K1 (phylloquinone) and vitamin K2 (menaquinones), we tested VK1 and VK2 separately to determine whether the observed microbial response was vitamer‐specific. At 10 µm, VK1 markedly inhibited the growth of *C. comes*, whereas the ethanol vehicle did not significantly affect growth relative to the untreated control (Figure 7a). AUC analysis confirmed a significant reduction following VK1 treatment compared with the vehicle control (Figure 7b). In contrast, VK2 produced a more moderate change in the growth curve and did not significantly alter the AUC. These findings suggest that the observed vitamin K–*C. comes* association may be primarily driven by VK1 under the tested conditions.

**FIGURE 7:**
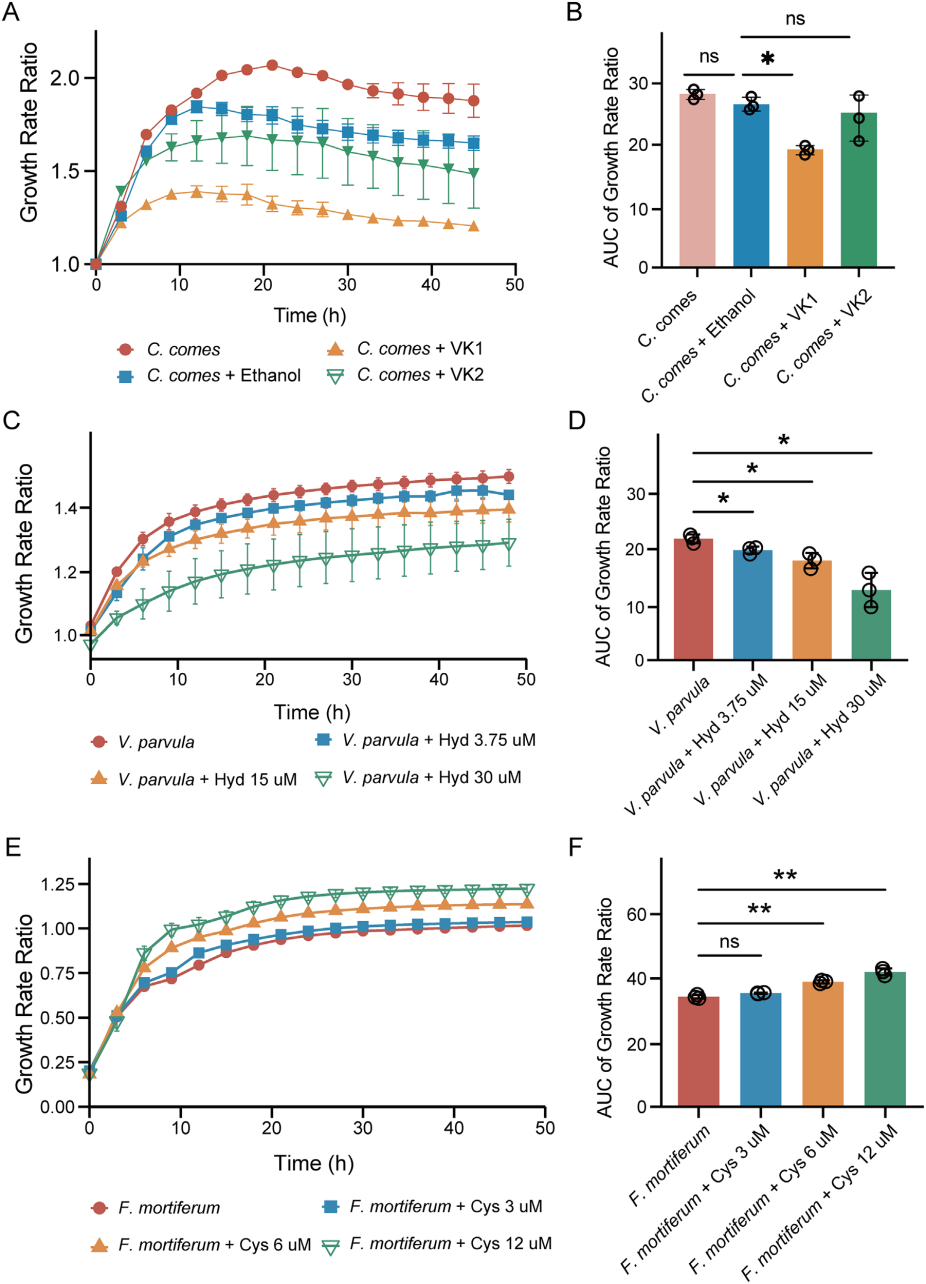
Experimental validation of selected metabolite‐microbe relationships. (a, c, e) Growth curves of *C. comes* cultured with vitamin K1 (VK1), vitamin K2 (VK2), ethanol (solvent control), or medium alone (control) (a); *V. parvula* cultured with 0, 3.75, 15, or 30 µm hydroxyproline (c); and *F. mortiferum* cultured with 0, 3, 6, or 12 µm cysteine (e) over 48 h. (b, d, f) Comparison of the area under the curve (AUC) among the different treatment groups for *C. comes* (b), *V. parvula* (d), and *F. mortiferum* (f). Data are presented as mean ± SD (n = 3). Each treatment concentration was compared with the untreated control using two‐sided *t*‐tests followed by Benjamini–Hochberg correction. ^*^, p < 0.05; ^**^, p < 0.01; ns, not significant.

We next examined the predicted negative association between hydroxyproline and *Veillonella parvula*. Hydroxyproline progressively reduced *V. parvula* growth at concentrations of 3.75, 15, and 30 µm, with the strongest inhibition observed at 30 µm (Figure 7c). AUC values were significantly lower at all tested concentrations compared with the untreated control (Figure 7d), indicating a concentration‐dependent inhibitory effect of hydroxyproline on *V. parvula* growth.

Conversely, we evaluated the predicted positive association between cysteine and *Fusobacterium mortiferum*. Cysteine supplementation promoted the growth of *Fusobacterium mortiferum* in a concentration‐dependent manner (Figure 7e). Although 3 µm cysteine did not significantly change the AUC, treatment with 6 or 12 µm cysteine significantly increased the AUC compared with the untreated control (Figure 7f). These findings support the direction of the association predicted by the MR analysis.

To further characterize the genomic features of the tested strains, we performed targeted eggNOG functional annotation analysis (Figure S13). *C. comes* harbored quinone/redox‐associated annotations relevant to vitamin K‐responsive physiology. *V. parvula* lacked annotated hydroxyproline‐specific degradation genes but retained general proline metabolism‐related functions. *F. mortiferum* contained multiple cysteine metabolism‐related annotations consistent with sulfur amino acid utilization. These annotations provide complementary genomic context but do not establish the molecular mechanisms underlying the observed metabolite‐dependent growth responses.

## 3 Discussion

This study represents a comprehensive investigation into the role of SVs in shaping the gut microbiome, blood metabolites, and their interactions. Through high‐depth whole‐genome sequencing of 2002 individuals, we identified a catalog of 138 859 high‐confidence SVs, a substantial proportion of which were not previously reported, thereby providing a valuable population‐specific genomic resource for the Chinese population. By integrating SV‐based association analyses with microbiome and metabolomic data, we identified 30 microbiome‐associated SVs and 48 metabolite‐associated SV associations, underscoring the critical and underexplored role of SVs in host‐microbiome‐metabolome crosstalk [26, 28, 78].

### 3.1 Novel Associations and Complementary Genetic Information

Our findings not only replicate but also significantly extend previous observations. For instance, beyond confirming the established link between the *HBA1/HBA2/HBM/HBQ1* deletion and red blood cell indices [37, 38, 79], we uncovered its novel associations with trace elements like copper and zinc, hinting at broader metabolic implications. Similarly, we identified a deletion in the haptoglobin gene associated with total cholesterol, corroborating prior reports [80], and novel links such as an insertion in the *TOX2 *gene [81] with lipid profiles, suggesting a potential nexus between genetic variation, metabolism, and immune regulation. More importantly, we discovered population‐specific signals, most notably an Asian‐specific 6.5‐kb deletion in the *TENT2 *gene strongly linked to* Coprobacillus sp. 29_1* abundance. Given *TENT2*’s role in miRNA adenylation [82] and miRNA's capacity to modulate gut microbiota [83], this axis presents a compelling, hypothesis‐generating link between host genetics, gene expression regulation, and the microbiome that warrants future functional validation. Furthermore, our data reinforce the role of SVs in the amylase (*AMY*) gene regions in modulating starch‐degrading microbes and pathways [25], illustrating how host genetic adaptation may operate through the microbiome [84].

Beyond identifying individual associations, our conditional analyses demonstrated that several SV associations remained significant after accounting for independent local SNP signals. At the community level, variance‐partitioning analyses further demonstrated that microbiome‐associated SVs contributed additional but modest variation beyond prioritized SNP signals. Although SNPs explained a larger proportion of microbiome variation overall, SVs retained an incremental contribution, supporting that SVs may represent a complementary genetic component that is not fully captured by SNP‐centric GWAS [85, 86, 87]. This finding underscores the necessity of incorporating SVs into future genetic studies of complex traits, including those involving the microbiome.

### 3.2 Advancing From Associations to MR‐Inferred Links and Experimental Support

A pivotal advancement of our work lies in moving beyond observational correlations. By employing SVs as instrumental variables, we applied MR to infer putative directional relationships, including relationships between *Clostridium ramosum* and serum uric acid, *Citrobacter* and 1‐methylhistidine, and vitamin K and *Coprococcus comes*. The robustness of these MR‐based directional inferences was significantly strengthened through multi‐tier validation: (1) the use of independent and strong instruments (F‐statistic > 10), (2) sensitivity analysis via two‐sample MR, and (3) crucially, in vitro experimental confirmation of the predicted inhibitory effect of vitamin K1 on *Coprococcus comes*, hydroxyproline reduced *Veillonella parvula* growth in a concentration‐dependent manner, and cysteine supplementation promoted *Fusobacterium mortiferum* growth under the tested conditions. This integrated approach provides a more rigorous framework for dissecting host‐microbe‐metabolite interactions than association studies alone.

### 3.3 Limitations and Future Directions

Several limitations should be considered. First, SV detection was conducted with short‐read sequencing data. Due to the nature of short‐read sequencing technology, although we employed the GATK‐SV pipeline and benchmarked with high‐confidence SV sets of NA12878 and the Chinese father genome from the Quartet Project, the false discovery rates (FDR) of DUP and INS can still affect GWAS robustness. Therefore, long‐read sequencing validation of SV calls and external validation of GWAS results are required in future studies. Second, the functional mechanisms underlying the majority of identified SV associations remain to be elucidated and require dedicated experiments. Third, MR analyses should be interpreted cautiously. Because exposure and outcome GWAS summary statistics were generated from the same cohort, the summary‐statistics‐based MR analyses represent sensitivity analyses rather than independent validation. In addition, many exposure–outcome pairs were supported by a limited number of instruments, restricting the application of some pleiotropy‐sensitive approaches. Larger independent cohorts and functional studies will be necessary to further evaluate these inferred relationships. Fourth, although individuals with antibiotic exposure within three months before fecal sampling were excluded and the mbGWAS models adjusted for measured demographic, gastrointestinal, dietary, and lifestyle covariates, the cross‐sectional design cannot fully exclude residual environmental confounding. Unmeasured or incompletely measured exposures, including medication history, short‐term dietary variation, living conditions, and socioeconomic factors, may influence gut microbial composition and could contribute to some of the observed associations. Finally, although this cohort represents one of the largest SV‐based microbiome studies to date, validation in populations with diverse genetic backgrounds and environmental exposures will be essential to determine the generalizability of these findings.

Future studies integrating high‐quality SV maps from long‐read sequencing, multi‐omics molecular profiling, and functional experiments will provide deeper insights into how host structural variation contributes to microbial ecology and systemic metabolism.

## 4 Conclusion

In summary, this study established a reference SV dataset for the Chinese population and demonstrates that host structural variation represents an important but previously underexplored genetic layer influencing gut microbiome composition and circulating metabolic traits. By integrating SV association analyses, genetic prioritization approaches, and targeted experimental evaluation, our work provides a framework for understanding how human genomic diversity contributes to host–microbiome–metabolome interactions.

## 5 Methods

### 5.1 Study Cohort and Samples

Samples used in this study were obtained from the 4D‐SZ omics cohort, a cohort that has been described elsewhere [14, 19, 20, 39, 40, 41, 88]. In the year 2017, a total of 2002 individuals provided blood samples, and 1539 of these individuals also provided matched fecal samples as part of the annual health check program. The blood samples were subjected to high‐depth whole‐genome sequencing and blood metabolic traits testing, whereas the fecal samples were subjected to shotgun metagenomic sequencing. For microbiome analyses, individuals who reported antibiotic exposure within three months prior to sampling were excluded. The collection protocols for blood and stool samples, as well as the sequencing procedures, were consistent with those used in earlier studies. Buffy coats were isolated from blood samples, and DNA was extracted using the HiPure Blood DNA Mini Kit (Magen, catalog no. D3111) according to the manufacturer's protocol. Fecal samples were collected using the MGIEasy kit, and stool DNA was extracted using the MetaHIT protocol. Subsequently, DNA concentrations were quantified using Qubit (Invitrogen), and 200 ng of DNA from each sample was used for library preparation. Paired‐end 100 bp and single‐end 100 bp sequencing were performed for blood and stool samples, respectively, on the BGISEQ‐500 platform [89].

Ethical approval for the study was obtained from the Institutional Review Boards (IRB) of BGI‐Shenzhen, and all participants provided written informed consent.

### 5.2 High‐Depth Whole Genome Sequencing and SNP/INDEL Detection

A total of 2002 individuals in this study were sequenced to a mean sequencing depth of 42× for the whole genome. The reads were aligned to the human reference genome GRCh38/hg38 using BWA (v.0.7.15) [90] with the default parameters. The adapter sequences and bases with quality values below five were removed. The alignments were indexed in the BAM format using Samtools (v.0.1.18) [91], and PCR duplicates were marked for downstream filtering using Picardtools (v.1.62). The Genome Analysis Toolkit's (GATK v.4.3 [92]) BaseRecalibrator created recalibration tables to screen known SNPs and insertions or deletions (INDELs) in the BAM files from dbSNP (v.150). GATKlite (v.2.2.15) was used for the subsequent base quality recalibration and removal of read pairs with improperly aligned segments as determined by Stampy. GATK's HaplotypeCaller was used for variant discovery. GVCFs containing SNVs and INDELs from GATK HaplotypeCaller were combined (CombineGVCFs), genotyped (GenotypeGVCFs), variant score recalibrated (VariantRecalibrator), and filtered (ApplyRecalibration). During the GATK VariantRecalibrator process, we took our variants as inputs and used four standard SNP sets to train the model: (1) HapMap3.3 SNPs; (2) dbSNP build 150 SNPs; (3) 1000 Genomes Project SNPs from the Omni 2.5 chip and (4) 1000 G phase1 high‐confidence SNPs. The sensitivity thresholds of 99.9% to SNPs and 99% to INDELs were applied for variant selection after optimizing for Transition to Transversion (TiTv) ratios using the GATK ApplyRecalibration command.

A stringent inclusion threshold for SNP/INDEL variants was established: (i) mean depth >8×; (ii) Hardy–Weinberg equilibrium (HWE) *p* > 10^−5^; and (iii) genotype calling rate > 98%. Furthermore, the sample criteria were as follows: (i) mean sequencing depth > 20×; (ii) variant calling rate > 98%; (iii) no population stratification by performing principal components analysis (PCA) analysis implemented in PLINK (v1.9) [93]; and (iv) excluding related individuals by calculating pairwise identity by descent (IBD, Pi‐hat threshold of 0.1875) in PLINK. Only ten samples were removed after quality control filtering. After variant and sample quality control, 1992 individuals with 8.5 million common (MAF ≥ 1%) SNP/INDEL variants were left for association analyses.

### 5.3 SV Detection

Due to the complexity of SV detection from short‐read sequencing data, the use of multiple SV calling tools is a common practice to promote accuracy and sensitivity. For SV detection, a pipeline was designed according to the SV‐Adjudicator [43] (https://github.com/talkowski‐lab/SV‐Adjudicator) and GATK‐SV cohort mode (https://github.com/broadinstitute/gatk‐sv). In the pipeline, the BAM file is obtained from the preceding alignment and filtering process as its primary input. Subsequently, Manta (v1.6.0) [94], Wham (v1.7.0) [95], Svaba (v1.1.3) [96], and Delly (v0.8.7) [97] were run for SV detection; xTea [98] was run for mobile element insertion discovery, and cn.MOPS (v1.40.0) [99] and GATK gCNV [100] were run for CNVs calling (Figure S3). The algorithms were selected according to Kosugi et al. [45]. Finally, the SV calls from the five algorithms and the CNV calls of each sample were standardized and integrated using the GATK‐SV pipeline. All computations were completed locally.

### 5.4 Sample Batching and Quality Control for SV

Following the batching strategy delineated by Collins et al. [42], all samples that passed the quality control (QC) were subdivided into five batches, with ∼380 samples in each batch according to the dosage score (WGD). The distribution of female and male samples in each batch was maintained at a ratio of approximately 1:1, ensuring balance across all batches.

For filtering after GATK‐SV integration, outlier samples exhibiting either fewer or higher‐than‐average SV calls per contig for each caller, or samples manifesting autosomal aneuploidies based on the per‐batch binned coverage of each chromosome, were excluded. Additionally, individuals with missing data or those who failed the preceding SNP/INDEL filtering were omitted. Finally, 1839 samples remained in the final SV callset.

### 5.5 SV Filtering

To curate a high‐quality SV dataset for subsequent genetic analyses, further filtration of the SVs was conducted post‐GATK‐SV pipeline execution and sample quality assessment. SVs with quality scores (GQ) > 250 or marked as “PASS” were retained, while variants of type “BND” were excluded. SVs with a genotyping rate >95% and Hardy–Weinberg equilibrium *p*‐value > 1 × 10^−6^ were kept. For GWAS analysis, only SVs with MAF >1% were retained, resulting in a final set of 22 519 SVs left for association analysis.

### 5.6 SV Annotation

The SVs that passed the quality control were annotated using GATK SVAnnotate (GATK v4.6.0) for functional prediction. The protein‐coding GTF file was downloaded from GENCODE (hg38.v37). Only the protein‐coding genes were retained for annotation. The BED files of the non‐coding regions were downloaded from GATK‐SV. AnnotSV (v3.3.8) was applied for additional SV annotation, utilizing the GRCh38 database build to incorporate information such as the pLI score of disrupted genes.

### 5.7 Validation of SV Using NA12878

To validate the SV detection process, the NA12878 human genome reference standard sequenced on the BGISEQ‐500 platform (https://db.cngb.org/search/experiment/CNX0094812/) was added as a demo into the cohort for GATK‐SV integration. To evaluate the SV detection, the final SV set of the demo was compared with the reference SV sets of NA12878 constructed with the datasets from the Database of Genomic Variants (http://dgv.tcag.ca/dgv/app/home) and the long‐read‐derived SV data [45]. The evaluation was performed using EvalSVcallers (https://github.com/stat‐lab/EvalSVcallers).

### 5.8 Validation of SV Using Chinese Trio

To further validate the SV detection process in an East Asian cohort context, the father sample from The Quartet Project, standard‐sequenced on the BGISEQ‐500 platform, was added as a Chinese population demo for GATK‐SV detection. To evaluate the SV detection, the final SV set of the demo was compared with the high‐confidence SV set of the Chinese father genome constructed with the datasets from The Quartet Project (https://chinese‐quartet.org/#/dashboard). The evaluation was also performed using EvalSVcallers as previously described. Due to the limitation of the reference dataset, the duplications were evaluated as insertions.

### 5.9 Comparisons With Published SV Datasets

We examined whether the SVs detected in this study have been reported in five publicly available SV databases, including gnomAD, Audano et al., 2019, HGSVC2, Wu et al., and dbVar. The gnomAD project is a high‐coverage (average depth of 32X) short‐read sequencing (SRS) dataset comprising 14 891 global samples. The dbVar database catalogs SVs from multiple platforms. We first ensured that the coordinates of the SVs in these public databases were in GRCh38. To compare our SVs with those in the public databases, we performed pairwise overlap analysis. For each pair of SV calls, we required an overlap rate ≥ 50% with our identified SV interval. For insertions, we also evaluated breakpoint distance tolerance: two INS were considered overlapping if their breakpoint distance was ≤ 20 bp. SVs that matched any entry in any of the five databases were defined as “known”; the remaining SVs were defined as “not reported”.

### 5.10 Shotgun Metagenomic Sequencing and Profiling

For fecal samples obtained from the SZ‐4D cohort, DNA libraries were constructed and sequenced using the BGISEQ‐500‐DNA sequencer for subsequent metagenomic analysis. Raw data were subjected to quality control and adapter trimming using fastp (v0.23.2) software. To filter out human reads, sequencing reads were aligned to the hg38 reference genome using SOAP2 (v2.22 [101]) with an identity threshold of ≥ 0.9. The gene profiles were generated by aligning high‐quality sequencing reads to the integrated gene catalog (IGC) using SOAP2 (identity ≥ 0.95), as previously described [102]. The relative abundance profiles of phylum, order, family, class, genera, and species were determined from the gene abundances. To eliminate the influence of sequencing depth in the comparative analyses, we downsized the unique IGC mapped reads to 20 million for each sample. The relative abundance profiles of genes, phyla, orders, families, classes, genera, and species were determined accordingly using the downsized mapped reads per sample.

Gut metabolic modules (GMMs) characterize the bacterial and archaeal metabolism specific to the human gut, emphasizing anaerobic fermentation processes [103]. The current GMM set was developed through a comprehensive review of the literature and metabolic databases, including MetaCyc [104] and KEGG, followed by expert curation to define modules and alternative pathways. Ultimately, we identified 616 common microbial taxa and GMMs present in at least 10% of the samples.

### 5.11 Microbiome Association Analysis With Common SVs

We examined the relationship between common host SVs (MAF ≥ 1%) and gut taxa/GMMs by utilizing linear or logistic models based on the abundance of each taxon/GMM. The abundance of taxon/GMM with an occurrence rate exceeding 95% in the cohort was log‐transformed, and outliers (deviating over four standard deviations from the mean) were excluded to ensure a reliable quantitative trait analysis. Alternatively, the taxon/GMM was categorized into a presence/absence pattern to mitigate zero inflation issues, treating the abundance as a dichotomous trait. Subsequently, for SNP/INDEL and SV identified in this cohort, we conducted a standard single variant‐based mbGWAS analysis using PLINK (v1.9) [93] with a linear model for quantitative traits or a logistic model for dichotomous traits.

To minimize potential environmental confounding, covariates were selected based on their reported associations with gut microbiome variation and the availability of phenotype information in this cohort. We first evaluated the contribution of demographic, anthropometric, lifestyle, and gastrointestinal‐related variables to microbiome variation. The final mbGWAS models included age, sex, BMI, defecation frequency, stool form, 12 diet and lifestyle variables (smoking, daily_sleep_hrs, mental pressure status, siesta, stay up frequency, dietary preference, yoghurt freq, soybean freq, milk freq, fruit freq, veg freq, and high sugar/fat freq), together with the first ten genetic principal components to account for population structure. Collectively, these covariates explained 2.76% of gut microbiome variation at the species level (Table S7).

### 5.12 Microbiome Association Analysis With Rare SVs

The SV burden test was conducted via a generalized linear regression (glm) model, adjusting for age, gender, BMI, defecation frequency, stool form, 12 diet/lifestyle factors, along with the top ten PCs as covariates. The gene‐based sequence kernel association test (SKAT) analysis was performed in rare SVs (MAF < 0.01) using the SKAT (v2.2.4) package in R with method = ‘skato’. Age, gender, BMI, defecation frequency, stool form, 12 diet/lifestyle factors, and the top ten PCs were added into the model as covariates. The rare SVs were filtered using plink v1.9 with options ‘–hwe 1e‐4 –max‐maf 0.01 –geno 0.02 –mind 0.02’. The gene‐based SV annotation was performed as described in the SV Annotation section. An SV was annotated within the gene if it overlapped with the gene region or was predicted to affect the promoter of the gene.

### 5.13 Metabolic Trait Profiling

In this study, metabolic traits, including anthropometric characteristics and blood metabolites, were assessed in all participants during physical examinations. Anthropometric data, including height, weight, waist circumference, and hip circumference, were recorded by nurses, while age and gender were self‐reported. Clinical evaluations, such as blood tests and urinalysis, were conducted by a certified physical examination organization. Hormones were measured in a similar manner, with 250 µL of plasma and atmospheric pressure chemical ionization (APCI). Trace elements were analyzed using a 7700x ICP‐MS (Agilent Technologies) equipped with octupole reaction system (ORS) technology to reduce spectral interferences, using 200 µL of whole blood. Water‐soluble vitamins were assessed by UPLC coupled to an Xevo TQ‐S Triple Quad mass spectrometer (Waters) with ESI in positive ion mode, utilizing 200 µL of plasma. Fat‐soluble vitamins were measured with UPLC linked to an AB Sciex Qtrap 4500 mass spectrometer using APCI and 250 µL of plasma. Blood amino acids were quantified using ultra‐high pressure liquid chromatography (UHPLC) coupled with an AB Sciex Qtrap 5500 mass spectrometer, utilizing electrospray ionization (ESI) in positive ion mode with 40 µL of plasma. Hyperuricemia is defined as an elevated serum uric acid level, with greater than 6 mg/dL in women and 7 mg/dL in men.

### 5.14 Metabolites Association Analysis

The log10‐transformed median‐normalized values of 121 anthropometric and metabolic traits were used as quantitative traits. Samples with missing values or values greater than 4 standard deviations from the mean were excluded from the association analysis. Each of the common SNP/INDEL or common SV (MAF ≥ 1%) was independently tested using a linear model for quantitative traits in PLINK v1.9. The gene‐based sequence kernel association test (SKAT) analysis was performed for rare SVs (MAF < 0.01) using the SKAT (v2.2.4) package in R, as done in the microbiome association analysis. Consistent with mbGWAS analyses, covariates included age, sex, BMI, defecation frequency, stool form, 12 diet/lifestyle factors, and the top ten genetic principal components.

### 5.15 Gene Enrichment Analysis

The genes harboring significantly associated SVs were annotated using AnnotSV [62, 63, 64]. Gene Ontology (GO) and Kyoto Encyclopaedia of Genes and Genomes (KEGG) gene functional enrichment analysis of genes harboring significantly associated SVs was executed using the R packages including clusterProfiler, enrichplot, ggplot2, and org.Hs.eg.db. A standardized metric (*p*‐value < 0.05, Q‐value < 0.2) was used to prioritize the top functional items and pathways. Biological process (BP), cellular component (CC), and molecular function (MF) were the three categories included in GO analysis.

### 5.16 Observational Correlation Analyses

All microbial features and metabolic traits were transformed using log‐transformed median‐normalized function to reduce the skewness of the distributions. The observational correlations between these variables were evaluated using multivariable linear regression analysis while adjusting for age and gender. The beta coefficient and *p*‐value were obtained.

### 5.17 One‐Sample MR Analysis

To explore the potential causal links between microbial traits and metabolic characteristics within the same cohort, we conducted a one‐sample bidirectional MR analysis in this cohort with both metabolite and microbiome data. We selected SV instruments based on a stringent genome‐wide significance threshold (*p* < 2.22 × 10^−^^6^) and performed clumping with an LD r^2^ < 0.1 to ensure independence among genetic variants for MR analysis. An unweighted polygenic risk score (PRS) was calculated for each participant based on independent genetic variants derived from GWAS data. For each SV, we coded the risk alleles as 0, 1, or 2, depending on the number of trait‐specific risk alleles carried by the individual. Instrumental variable (IV) analyses were conducted using two‐stage least squares (TSLS) regression. In the first stage, we assessed the association between the PRS and the observational phenotype using linear regression, from which we derived fitted values representing the predicted exposure. During this process, the *F*‐statistics and the variance explained by IVs were calculated. We required the *F*‐statistics to be over 10 to ensure a strong IV for analysis. In the second stage, we regressed the outcome trait on the genetically predicted exposure from the first stage. Both stages were adjusted for potential confounders, including age, gender, and BMI. TSLS was performed using the ‘ivreg’ function from the AER package in R (v.1.2‐9; https://www.rdocumentation.org/packages/AER/versions/1.2‐9/topics/ivreg).

### 5.18 Summary‐Statistics‐Based Sensitivity Analysis

We conducted two‐sample MR analyses as a summary‐statistics‐based sensitivity analysis to assess the directionality of associations between microbial features and blood metabolic traits. Summary statistics from the corresponding GWAS analyses were used for the MR analyses. Two complementary MR analytical strategies were implemented depending on the number of available instrumental variables (IVs). When only one IV was available, the Wald ratio method was used; when more than one IV was available, the inverse‐variance weighting (IVW) method was used as the primary analysis. All analyses were conducted using the “TwoSampleMR” package in R. SVs associated with each exposure were selected as IVs at a significance threshold of *p* < 2.22 × 10^−^^6^, and linkage disequilibrium (LD) pruning was applied to remove correlated variants (r^2^ < 0.1). The MR results were interpreted as evidence of potential directional associations rather than definitive causal effects.

### 5.19 In Vitro Growth Inhibition Assay

To evaluate selected metabolite‐microbe relationships prioritized by the MR analyses, growth kinetic assays were performed in 96‐well microplates. Coprococcus comes AM115‐28, Veillonella parvula AF120‐66, and Fusobacterium mortiferum AM25‐18LB were obtained from an in‐house human gut bacterial strain collection comprising more than 20,000 bacterial isolates cultured from fecal samples of healthy individuals, as previously described [105]. Exponential‐phase bacterial cultures were diluted in the appropriate growth medium to an initial OD600 of approximately 0.2. *Coprococcus comes* AM115‐28 was cultured in brain heart infusion (BHI) medium, whereas *Veillonella parvula* AF120‐66 and *Fusobacterium mortiferum* AM25‐18LB were cultured in de Man, Rogosa and Sharpe (MRS) medium. Vitamin K1 (Phylloquinone) and Vitamin K2 (Menaquinone‐4) were purchased from Macklin. Each condition was tested in triplicate. Unless otherwise specified, the strains were cultured at 37°C under strict anaerobic conditions consisting of 90% N_2_, 5% CO_2_, and 5% H_2_ in an anaerobic chamber (Bactron IV‐2, Shellab, USA).

For the vitamin K experiment, *C. comes* was assigned to four groups: (i) an untreated control containing inoculated BHI medium; (ii) a vehicle control containing 0.1% (v/v) absolute ethanol; (iii) a VK1 group containing 10 µm vitamin K1 dissolved in ethanol; and (iv) a VK2 group containing 10 µm vitamin K2 dissolved in ethanol. The final ethanol concentration was matched among the vehicle, VK1, and VK2 groups. Uninoculated BHI, BHI plus ethanol, BHI plus VK1, and BHI plus VK2 wells were included as background and sterility controls.

For the hydroxyproline experiment, *V. parvula* was cultured in MRS medium containing 0, 3.75, 15, or 30 µm hydroxyproline. For the cysteine experiment, F. mortiferum was cultured in MRS medium containing 0, 3, 6, or 12 µm cysteine. Uninoculated MRS wells were included on each plate for background and sterility monitoring.

Plates were incubated under anaerobic conditions at 37°C with continuous low‐speed shaking at 60 rpm. OD600 was measured every 3 h for 48 h using a Varioskan LUX multimode microplate reader (Thermo Fisher Scientific) controlled with SkanIt Software v7.0.2. For the vitamin K experiment, OD600 values were normalized to the corresponding 0‐h baseline to generate growth ratios. For the hydroxyproline and cysteine experiments, a single common normalization constant was calculated separately for each bacterial experiment as the pooled mean 0‐h OD600 across the four included groups and their three replicate wells (12 baseline measurements in total). Every OD600 measurement from the corresponding experiment was divided by this same constant to generate growth ratios, thereby retaining the relative differences among treatment groups.

The area under the curve (AUC) was defined as the time integral of the normalized growth‐ratio curve from 0 to 48 h and was calculated using the trapezoidal rule based on measurements collected at 3‐h intervals. Thus, the AUC represents cumulative normalized bacterial growth over the entire 48‐h incubation period. For each experiment, the AUC at each non‐zero treatment concentration was compared with that of the corresponding untreated control using a two‐sided Welch's t‐test. The resulting p‐values were adjusted within each experiment using the Benjamini–Hochberg method. Data are presented as mean ± s.d., and an adjusted *p* < 0.05 was considered statistically significant.

## Author Contributions

X.L and T.Z. conceived and organized this study. X.X., X.J., and L.X. led the organization of the cohort, the sample collection, DNA extraction and sequencing, and the questionnaire collection. X.L and K.W. processed the whole‐genome data. X.L., Q.Z., and J.C. processed the metagenome data. X.L. and Q.Z. performed the metagenome‐genome‐wide association analyses and Mendelian randomization analyses. H.W. and Y.Z. performed the in vitro experimental validation. X.L. wrote the raw manuscript. All authors contributed to data and text in this manuscript.

## Conflicts of Interest

The authors declare no conflicts of interest.

## Supporting information




**Supporting File 1**: advs78201‐sup‐0001‐SuppMat.docx.

**Supporting File 2**: advs78201‐sup‐0002‐TableS1.xlsx.

## Data Availability

All summary statistics that support the findings of this study, including the population SV dataset, were available in the Supplementary Tables. The gut metagenomic sequencing data after removing host reads have been publicly available in the GSA at https://ngdc.cncb.ac.cn/gsa‐human/browse/HRA000907. All the metabolites and individual‐level host genetics data have been uploaded to the GSA database (https://ngdc.cncb.ac.cn/bioproject/browse/PRJCA005334). According to the Human Genetic Resources Administration of China regulation and the institutional review board of BGI‐Shenzhen related to protecting individual privacy, these data are controlled‐access and are available via an application on request. The human reference hg38 datasets are publicly available from http://hgdownload.soe.ucsc.edu/goldenPath/hg38/bigZips/. The datasets of common SVs are available at the following link: GnomAD SV: https://datasetgnomad.blob.core.windows.net/dataset/release/4.1/genome_sv/gnomad.v4.1.sv.sites.vcf.gz, dbvar SV: https://gnomad.broadinstitute.org/, HGSVC SV: http://ftp.1000genomes.ebi.ac.uk/vol1/ftp/data_collections/HGSVC2/release/v1.0/integrated_callset/freeze3.sv.alt.vcf.gz, Wu et al. SV: https://ftp.ncbi.nlm.nih.gov/pub/dbVar/data/Homo_sapiens/by_study/csv/all_variants_for_nstd212.csv.gz, Audano et al. 2019:http://ftp.1000genomes.ebi.ac.uk/vol1/ftp/data_collections/hgsv_sv_discovery/working/20181025_EEE_SV‐Pop_1.
