# Supplementary material for: Structural Variants in the Human Genome Associated With Gut Microbiome and Blood Metabolic Traits With Potential Causal Links
Source: Adv Sci (Weinh). 2026 Oct 11:e78201. Online ahead of print. doi: 10.1002/advs.78201 (PMC13655246; doi:10.1002/advs.78201)
Supplement: Supplementary file 1 [file ADVS-9999-e78201-s001.docx]

**Supplementary figure 1. Overview of the individuals from the 4D-SZ cohort in this study.**

Among the 2,002 samples, 53% were females. The average age of the cohort was 30 years, ranging from 21 years to 67 years.

**Supplementary figure 2. The distributions of sequencing depth (a) and base mapping rate (b) of the samples.**

(a) The plot illustrating the sequencing depth of the 2,002 samples. Each point represents a sample, with the total number of samples on the x-axis and mean sequencing depth on the y-axis. The dashed line represents the mean depth of 42x.

(b) Violin plot displaying the base mapping rate distribution of the 2,002 samples. The width of the violin plot represents the density of mapping rate values, with scatter points overlaid to indicate the individual mapping rate for each sample. The plot shows that the mapping rates are predominantly distributed within the 97%-100% range, with a concentrated distribution in the 99%-100% interval.

**Supplementary figure 3. Workflow of SV calling and filtering.**

The bam file of each sample was used for SV calling. Manta, Wham, Svaba, and Delly were run for SV detection, Xtea was run for mobile element insertion discovery, and cn.MOPS and GATK gCNV were run for CNVs calling. The SV evidences were collected through GATK CollectSVEvidence and GATK CollectReadCounts. The SV calls from the five algorithms and the CNV calls of each sample were standardized and integrated by GATK-SV pipeline.

**Supplementary figure 4. This scatter plot illustrates the correlation between the number of detected SVs and the sequencing depth across multiple samples.**

Each blue point represents an individual sample, the x-axis indicating the sequencing depth (in reads), the y-axis displaying the corresponding count of SVs identified. A red smoothing curve fitted using generalized additive models (GAM) is included to demonstrate the nonlinear trend between sequencing depth and counts of SVs. The shaded area represents the 95% confidence interval of the smoothing curve.

**Supplementary figure 5. Relationship between breakpoint distance threshold and the number of overlapping INS.**

The x-axis represents the breakpoint distance threshold (bp), ranging from 20 to 1,000 bp. The y-axis represents the number of overlapping insertion sequences (INS). As the threshold increases from 20 bp (15,129 overlapping INS) to 50 bp (18,526), 100 bp (20,766), 200 bp (25,676), 500 bp (35,714), 750 bp (41,920), and 1,000 bp (46,915), the number of overlapping INS shows a monotonic increasing trend.

**Supplementary figure 6. This scatter plots illustrates the correlations between SV burdens and α-diversity indices, namely Shannon index (a) and Simpson index (b), respectively.**

The marked regression coefficients (β) and *p-*values were obtained from the linear regression analysis adjusting for sex and age.

**Supplementary figure 7. The Manhattan plots of SV with α-diversity indices, Shannon index and Simpson index, respectively.**

**Supplementary figure 8. The boxplots illustrate the correlation between the *TENT2* DEL and four metabolites.** The marked *p*-values were from the metabolic GWAS analysis.

**Supplementary figure 9.** **The functional annotation of significantly associated SVs with the gut microbiome.**

GO (**a**) and KEGG analysis (**b**) for genes near SVs significantly associated with the gut microbiome. GO and KEGG pathway analyses were conducted, prioritizing significant functional items and pathways based on a standardized metric (*p* < 0.05, Q < 0.2). Comparison of *p-*values associated with the gut microbiome between SVs and nearby SNPs.

**Supplementary figure 10.** **The distribution of genomic inflation factor (λ) for GWAS tests of 121 blood metabolic traits.**

The λ values ranged from 0.80 to 1.33 with a median value of 1.

**Supplementary figure 11. The comparison of the associations between SVs and their nearby SNPs with blood metabolites.**

The plot highlights the association significance of 48 significant SV-metabolite associations and their nearby SNPs-metabolite associations. Extend 20000 bp on both sides of the existing SV range and identify SNP sites within this extended region as SNPs near the SVs. The x axis indicates the log10-transformed *p-*values. The y axis indicates the associations between the SVs and gut microbiome (N = 48). The red dots represent the *p-*values associated with SVs; the blue dots represent the *p-*values associated with SNPs.

**Supplementary figure 12. Instrument strength for microbiome features and blood metabolites.**

Box plots show the F-statistics (left panel) and explained variance (right panel) of instrumental variables for microbiome features and blood metabolites. This figure summarizes the distribution of instrument strength metrics across different exposures.

**Supplementary figure 13. eggNOG annotation counts for target KOs in selected metabolic pathways.**

Bar plots showing the number of eggNOG annotations (x-axis) matching target KEGG Orthology (KO) entries for three categories of metabolic pathways or enzyme classes. **(A)** Quinone-related pathways: Menaquinone biosynthesis (n = 0), Quinone-linked NADH-Q (n = 3), and Redox-protection proteins (n = 10). **(B)** Hydroxyproline and proline metabolism: Hydroxyproline degradation (M00948, 0/7 KOs), Hydroxyproline-specific enzymes (0/5 KOs), Proline biosynthesis (proB/proA/proC, 3/3 KOs), and Sodium/proline symporter PutP (K11928, n = 1). **(C)** Cystine-related metabolism: Cysteine biosynthesis (K00640/K01738/K01740, n = 2), C-S beta-lyase (K14155, n = 4), Cysteine desulfurase (K04487/K11717, n = 1), and Methionine gamma-lyase (K01761, n = 4). The horizontal bars and dots represent the annotation count for each pathway, with the number of target KOs or n values labeled on the right.
